# Effect of the coordination of π-acceptor 4-cyanopyridine ligand on the structural and electronic properties of *meso*-tetra(*para*-methoxy) and *meso*-tetra(*para*-chlorophenyl) porphyrin cobalt(ii) coordination compounds. Application in the catalytic degradation of methylene blue dye†

**DOI:** 10.1039/c9ra08504a

**Published:** 2020-02-14

**Authors:** Mouhieddinne Guergueb, Soumaya Nasri, Jihed Brahmi, Frédérique Loiseau, Florian Molton, Thierry Roisnel, Vincent Guerineau, Ilona Turowska-Tyrk, Kaïss Aouadi, Habib Nasri

**Affiliations:** University of Monastir, Laboratoire de Physico-chimie des Matériaux, Faculté des Sciences de Monastir Avenue de l'environnement 5019 Monastir Tunisia; Department of Chemistry, College of Science Al-Zulfi, Majmaah University Saudi Arabia soumaya.n@mu.edu.sa + 966 0164044044; Département de Chimie Moléculaire, Université Grenoble Alpes 301 rue de la Chimie, CS 40700 38058 Grenoble Cedex 9 France; Institute of Chemical Sciences of Rennes, UMR 6226, University of Rennes 1 Beaulieu Campus 35042 Rennes France; Institut de Chimie des Substances Naturelles CNRS Avenue de la Terrasse, F-91198 Gif-sur-Yvette France; Faculty of Chemistry, Wrocław University of Technology Wybrzeże Wyspiańskiego 27 50-370 Wrocław Poland; Department of Chemistry, College of Science, Qassim University Buraidah 51452 Saudi Arabia

## Abstract

To examine the influence of both the important π-acceptor character of the 4-cyanopyridine ligand and the nature of the *para*-substituted phenyls of *meso*-porphyrins on the electronic, electrochemical and structural properties of cobaltous metalloporphyrins, we prepared and fully characterized two coordination compounds: the (4-cyanopyridine)[*meso*-tetra(*para*-methoxyphenyl)porphyrinato]cobalt(ii) and the (4-cyanopyridine)[*meso*-tetra(*para*-chlorophenyl)porphyrinato]cobalt(ii) with the [Co^II^(TMPP)(4-CNpy)] and [Co^II^(TClPP)(4-CNpy)] formulas (complexes 1–2). The solution structures of compounds 1–2 were confirmed by ^1^H NMR spectroscopy and mass spectrometry methods. They were further characterized by cyclic voltammetry and photoluminescence studies. The X-ray molecular structure data show that the Co-TClPP-4-NCpy derivative (2) exhibits high ruffling deformation compared to that of the Co-TMPP-4-CNpy species (1). Notably, the crystal packing of complex 1 shows the formation of Co⋯Co supramolecular dimers with a distance of 5.663 Å. As an application of our two cobaltous compounds, an investigation involving complexes 1–2 in the degradation of the methylene blue dye in the presence and absence of H_2_O_2_ in aqueous solutions was carried out. These promising results show that 1–2 can be used as catalysts in the degradation processes of dyes.

## Abstract 2

Preparation and UV/vis, IR, MS, ^1^H NMR, cyclic voltammetry and molecular structures of two new Co(ii) complexes with *para*-methoxy-phenyl and *para*-chloro *meso*-porphyrins and 4-cyanopyridine ligand (1–2). Catalytic oxidation data of MB dye using 1–2.

## Graphical abstract

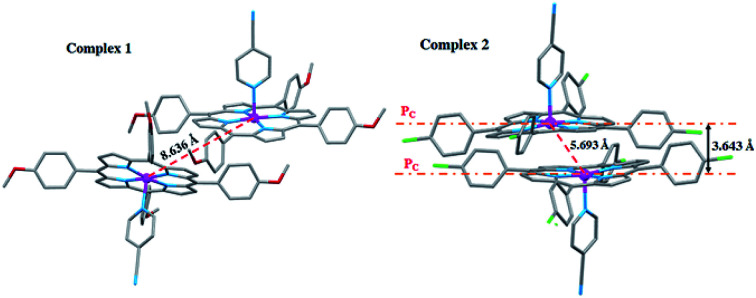

## 1. Introduction

The spectroscopic, structural, magnetic and electrochemical properties of metalloporphyrins involving several metal ions such as Fe(ii), Fe(iii), Zn(ii), Cd(ii), Sn(iv), Mg(ii), Mn(iii) and Co(ii) have been investigated by our group for more than two decades.^1–7^ The study of these tetrapyrrolic coordination compounds is of great importance because they provide good models for understanding the structural, electronic and magnetic properties of hemoproteins and heme-like enzymes such as cytochromes P450. Recently, porphyrins and porphyrin related compounds are finding crucial use in new applications such as catalysis in solar photovoltaic cells,^8–10^ as chemical sensors^11,12^ and as photosensitizing agents (PS) for photodynamic therapy (PDT).^13,14^ Notably, our research group has been involved in the preparation of catalytic compounds that can be used in the degradation and the removal of dyes used mainly in the printing industry.^15,16^ These colored species are an important component in causing water pollution.

It is noteworthy that the interaction of metalloporphyrins with neutral or anionic species *via* axial coordination plays an important role in the structural, electronic, magnetic and physical properties of these coordination compounds. The most studied of these species are the ones with iron and zinc as center metals. Cobalt metalloporphyrins have been less studied so far, but interest in them is growing and so are their reports in literature. Cobalt(ii) porphyrin complexes are paramagnetic and have the 3d^7^ electron configuration in the ground state. These cobaltous species are much more stable than the iron(ii) metalloporphyrins^17^ and are very easy to prepare compared to the ferrous metalloporphyrins. Early EPR investigations^16,18,19^ indicated that these Co(ii) species are low-spin (*S* = 1/2) in that the unpaired electron is located in the axial d_*z*^2^_ orbital (^2^A_1_ state). These electronic properties of cobaltous complexes is the principal reason why Co(ii) porphyrin complexes are increasingly investigated and their application fields are becoming wider.^7,20^

In continuation of our investigations on metalloporphyrins, we report herein the preparation and the characterization of two cobaltous porphyrin complexes involving the 4-cyanopyridine (4-CNpy) N-donor axial ligand and the two *meso*-porphyrins with either the phenyl *para*-substituted methoxy group or chlorine atom, namely the *meso*-tetra(*para*-methoxyphenyl)porphyrin (H_2_TMPP) and the *meso*-tetra(*para*-chlorophenyl)porphyrin (H_2_TClPP). The principal goal of this work is to study the electronic and the structural effects when the 4-cyanopyridine axial ligand, known to have a very important π-acceptor character, reacts with the two starting material tetracoordinated cobaltous complexes with a *meso*-porphyrinato *para*-substituted with a good donor OMe group (TMPP porphyrinato) and with a *meso*-porphyrinato *para*-substituted with the acceptor chlorine atom (TClPP moiety).

Many studies on the 4-cyanopyridine ion iron(iii) ion complex types [Fe^III^(Porph)(4-CNpy)_2_]^+^, using the following four *meso*-porphyrins: (1) the unsubstituted *meso*-tetraphenylporphyrin (H_2_TPP), (2) the methyl *para*-substituted *meso*-porphyrin (the *meso*-tertratolylphenylporphyrin H_2_TTP), (3) the 2,3,4-methyl *para*-substituted *meso*-porphyrin (the *meso*-tetramesitylporphyrin H_2_TMP) and (4) the OMe *para*-substituted *meso*-porphyrin (H_2_TMPP) have been carried out.^21,23^ For these ferric low-spin metalloporphyrins, the very high ruffling of the porphyrin is related to the important π-acceptor character of the 4-CNpy axial ligand. Thus, it has been shown that for three of the porphyrinato, *i.e.* TTP, TMP and TMPP, the ruffling deformation of the porphyrin core of these complexes are affected by the type of the phenyl-substitution of these three *meso*-porphyrins, unlike in the unsubstituted porphyrin derivative [Fe^III^(TPP)(4-CNpy)_2_]^+^. The second important goal of the present study is to test the efficiency of our two cobaltous species 1–2 in the adsorption and the catalytic oxidative degradation of the methylene blue dye in aqueous solutions.

## 2. Experimental section

### 2.1. Materials and methods

All reagents employed were commercially available and were used as received without further purification. The synthesis of the *meso*-tetra(*para*-methoxyphenyl)porphyrin (H_2_TMPP) and the *meso*-tetra(*para*-chlorophenyl)porphyrin (H_2_TClPP), the starting material complexes [Co^II^(TMPP)] and [Co^II^(TClPP)] were performed according to the methods previously described.^24,25^ All manipulations were carried out under aerobic conditions.

^1^H NMR spectroscopic characterization were performed with a Bruker DPX 400 spectrometer and chemical shifts (*δ*) are reported in ppm downfield from internal tetramethylsilane (TMS).

The UV-visible spectra were recorded with a WinASPECT PLUS (validation for SPECORD PLUS version 4.2) scanning spectrophotometer. Fourier-transform infrared spectroscopy (FT-IR) spectra were recorded on a PerkinElmer Spectrum Two FT-IR spectrometer.

Emission spectra were recorded in dichloromethane for complex 1 and in THF for complex 2 at room temperature on a Horiba Scientific Fluoromax-4 spectrofluorometer. Samples were placed in 1 cm path length quartz cuvettes. Luminescence lifetime measurements were performed after irradiation at wavelength values between 412 and 550 nm obtained by the second harmonic of a titanium:Sapphire laser (picosecond Tsunami laser spectra physics 3950-M1BB + 39868-03 pulse picker doubler) at an 800 kHz rate of repetition. For the decay acquisition, Fluotime 200 (AMS technologies) was utilized consisting of a GaAsmicro channel plate photomultiplier tube (Hamamatsu model R3809U-50) followed by a time-correlated single photon counting system from Picoquant (PicoHarp300). The ultimate time resolution of the system is close to 30 ps. Luminescence decays were analyzed with FLUOFIT software available from Picoquant. Emission quantum yields were determined at room temperature in dichloromethane solutions using the optically dilute method.^26^ [Zn(TPP)] in air-equilibrated dichloromethane solution was used as quantum yield standard (*ϕ*_f_ = 0.031). The instrumental uncertainties are as follows: absorption maxima 2 nm; molar absorption, 20%; emission maxima, 5 nm; emission lifetimes, 10%; emission quantum yields, 20%. The fluorescence quantum yield (*ϕ*_f_) was measured using the following equation (eqn (1)):^27^1
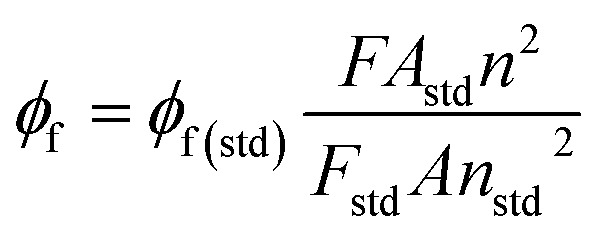
where *ϕ*_f(std)_ is the fluorescence quantum standard (in dichloromethane) (*ϕ*_f(std)_ = 0.03), the reference used is [Zn(TPP)] as it was mentioned above. *F* and *F*_std_ are the areas of the fluorescence emission plots for both the sample and the standard. *A*, *n*, *A*_std_ and *n*_std_^2^ are the relative absorbance of the sample and the refractive indices of the solvents for the sample and standard at the excitation wavelength.

Electrospray (ESI) spectra were obtained using an amaZon speed instrument. The samples were made by a 5 × 10^−3^ M in dichloromethane and the ESI-HSMS spectra were recorded in an LTQ Orbitrap XL (Thermo Scientific). The samples were made by 3 × 10^−3^ M in tetrahydrofuran (THF) then diluted to 5 × 10^−5^ M in methanol.

Electrochemistry: cyclic voltammetry (CV) experiments were performed with a CH-660B potentiostat (CH Instruments). All analytical experiments were conducted at room temperature under an argon atmosphere (argon stream) in a standard one-compartment, three-electrode electrochemical cell. Tetra-*n*-butylammonium perchlorate (TBAP) was used as the supporting electrolyte (0.2 M) in dichloromethane previously distilled over calcium hydride under argon. An automatic ohmic drop compensation procedure was systematically implemented before the CV data were recorded with electrolytic solutions containing the studied compounds at concentrations of *ca.* 10^−3^ M. CH Instruments vitreous carbon (*ϕ* = 3 mm) working electrodes were polished with 1 μm diamond paste before each recording. The Ag/AgNO_3_ 0.01 M (TBAP 0.2 in CH_2_Cl_2_) redox couple was used as the reference electrode. The potential of the ferrocene/ferrocenium redox couple was used as an internal reference (86 mV *vs.* Ag/AgNO_3_ under our experimental conditions). For comparison with previously published data, all potentials given in the text and in Table 5 have been converted to values relative to the saturated calomel electrode (SCE) by using the following relationship: *E*(SCE) = *E*(Ag/AgNO_3_) + 298 mV.

Adsorption and oxidation degradation of the methylene blue dye: the experiments were carried out at room temperature using 10 mg of complexes 1–2 and 5 ml of an aqueous solution of the methylene blue (MB) dye (at pH = 6). The agitation was kept at 150 rpm. The resultant mixture was filtrated and then the concentration was determined by measuring the absorption at 665 nm. The adsorbed amount *q*_*t*_ (mg g^−1^) was calculated according to the following formula (eqn (2)):

2 *Q* (mg g^−1^) = (*C*_o_ − *C*_*t*_) (V m^−1^)

where *C*_o_ and *C*_*t*_ are the dye concentration before and after the adsorption, respectively. *V* is the volume of the dye used and *m* is the mass of the adsorbent. The oxidation degradation of the MB dye using our cobaltous derivatives 1–2 as catalysts was performed using an aqueous solution of H_2_O_2_ (4 ml l^−1^). After filtration of the obtained mixture, concentration of the resultant solution was made by measuring the absorption at *λ*_max_ = 665 nm.

### 2.2. Synthesis of [Co^II^(TMPP)(4-CNpy)]·CHCl_3_ (1)

The [Co^II^(TMPP)] starting material (25 mg, 0.03 mmol) and 4-cyanopyridine (30 mg, 0.288 mmol) were dissolved in chloroform (5 ml). The reaction mixture obtained was stirred for 24 hours before it was overplayed by *n*-hexane. Dark purple crystals, suitable for X-ray diffraction, were obtained within 10 days, by slow diffusion of the *n*-hexane through the chloroform solution the yield is about 80%. Elemental analysis (%) calcd for C_55_H_41_Cl_3_CoN_6_O_4_ (1015.26). C, 65.07, H, 4.07, N, 8.28; found: C, 65.31, H, 4.21, N, 8.49. ^1^H NMR (400 MHz, CDCl_3_) *δ*(ppm), 14.64 (s, 8H β-pyrrole), 11.42 (s, 8H ArH) 9.01 (s, 2H, HL_2,6_), 8.87 (s, 8H, ArH), 7.98 (s, 2H, HL_3,5_), 5.01 (H–OCH_3_). UV-visible [*λ*_max_ (nm) in CH_2_Cl_2_ (*ε* × 10^−3^, mol^−1^ l cm^−1^)]: 437(420), 558(48), 600(35). FTR-IR (*ν̄* in cm^−1^): 3000–2830 *ν*[(CH) porphyrin], 994 [*δ*(CCH) porphyrin], 2237 [*ν*(C≡N) 4-CNpy]. HSMS (ESI^+^, CH_2_Cl_2_): *m*/*z* = 791.7714 [Co^II^(TMPP)]^+^.

### 2.3. Synthesis of [Co^II^(ClPP)(4-CNpy)] (2)

The preparation of the [Co^II^(TClPP)(4-CNpy)] (2) was performed by the same procedure as complex 1 using [Co^II^(TClPP)] (30 mg, 0.036 mmol) and the 4-cyanopyridine (30 mg, 0.288 mmol) reagents. Dark purple crystals, suitable for X-ray diffraction, were obtained within one week by slow diffusion of the *n*-hexane through the dichloromethane solution, the yield is about 85%. Elemental analysis (%) calcd for [Co^II^(TClPP)(4-CNpy)]·CH_2_Cl_2_ (we take into account the dichloromethane molecule omitted by the Squeeze-Platon program in solid state structure):

Elemental analysis (%) calcd for C_51_H_30_N_6_Cl_6_Co (998.49). C, 61.35, H, 3.03, N, 8.42; found: C, 61.87, H, 3.21, N, 8.50. ^1^H NMR (400 MHz, CDCl_3_) *δ* (ppm): 14.61 (s, 8H β-pyrrole), 11.53 (s, 8H ArH), 9.42 (s, 2H HL_2,6_), 9.01 (s, 8H, ArH), 8.81 (s, 2H HL_3,5_). UV-visible (CH_2_Cl_2_), [*λ*_max_ (nm) in CH_2_Cl_2_ (*ε* × 10^−3^, mol^−1^ l cm^−1^)]: 436(420), 556(49), 598(44) FTR-IR (*ν̄* in cm^−1^): 2970–2830 *ν*[(CH) porphyrin], 993 [*δ*(CCH) porphyrin], 2234 [*ν*(C≡N) 4-CNpy]. MS (ESI^+^, THF): *m*/*z* = 808.45 [Co^II^(TClPP)]^+^ found 808.98, *m*/*z* = 913.55 [Co^II^(TMPP)(4-CNpy)] found 913.04.

### 2.4. X-ray structure determination

The data collections for 1–2 were performed using a D8 VENTURE Bruker AXS and a Bruker-AXS-Enraf-Nonius Kappa APEXII diffractometers respectively. Both two diffractometers were equipped with a graphite-monochromator. The X-ray radiation used was the Mo-Kα radiation (*λ* = 0.71073 Å). The intensity data for 1–2 were collected by the narrow-frame method at low temperature. Both unit cells parameters for 1–2 were calculated and then refined from the full data collections. The reflections were scaled and corrected for absorption effects using the SADABS program version 2.10 (Bruker AXS 2001).^28^ Complex 1 presents four disordered problems; (i) one phenyl group of the TMPP porphyrinato is disordered in two positions (C28A–C29A–C30A–C31A–C32A–C33A and C28B–C29B–C30B–C31B–C32B–C33B) with practically the same occupancy factors (∼50%/50%), (ii) two methyl groups of two methoxy moieties in *para*-position of two phenyls of the TMPP porphyrinate present each one two positions (C34A/C34B and C41A/C41B) with occupancy values of ∼50%/50% and 80%/20% respectively and (iii) the chloroform solvent is also disordered in two positions with the major position C55A–Cl1A–Cl2A–Cl3A exhibits an occupancy of 60%. For the same complex 1, the SIMU constraint command was used to minimize the ADPs of several phenyl atoms of the TMPP porphyrinato. One phenyl ring of the TClPP porphyrinate of complex 2 is disordered in two positions (C34–C35–C37–C38/C34A–C35A–C37A–C38A) with occupancies of 0.63(1) and 0.29(1). For this compound, a dichloromethane molecule found to be badly disordered was removed using the PLATON SQUEEZE^29^ procedure. Therefore, the given chemical formula of 2 and other crystal data of this complex do not take into account the removed solvent molecule. The crystallographic data, the structural refinement details, and selected bond lengths and angles for 1–2 are reported in Tables SI-1 and SI-2.†

### 2.5. Hirshfeld surface analysis

The Hirshfeld surface analysis and the associated 2D fingerprint plots were used to quantify the intermolecular interactions in the crystal for complexes 1–2. Based on the crystallographic CIF (Crystallographic Information File) files of 1–2, the Crystal Explorer17.5 program has been utilized to obtain the Hirshfeld surfaces and the 2D fingerprint plots of our two cobaltous derivatives.^30^ Using equation (eqn (3)), the different normalized contact distances “*d*_norm_” were calculated where “*d*_e_” is the distance from the point to the nearest nucleus external to the surface, “*d*_i_” is the distance to the nearest nucleus internal to the surface and “vdW” is the van der Waals radii of the atom.^31^3
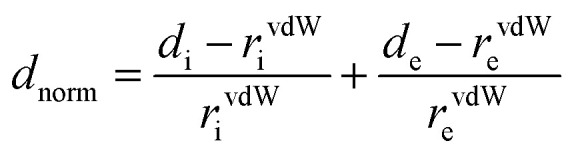


The *d*_norm_ values are displayed using a red-white-blue color scheme corresponding to negative values (red regions) for shorter contacts, zero (white regions) for contacts around the van der Waals separation and positive (blue regions) for longer contacts. On the other hand, the Hirshfeld surface *d*_norm_ appears with a red concave region around the acceptor atom and a corresponding blue region around the donor atom. The fingerprint plots appear where one molecule acts as a donor (*d*_e_ > *d*_i_) and the other as an acceptor (*d*_e_ < *d*_i_). The proportions of the intermolecular interactions are presented in a 2D fingerprint which is obtained by a combination of *d*_e_ and *d*_i_.^32^

## 3. Results and discussion

### 3.1. ESI mass studies

**Fig. 1:**
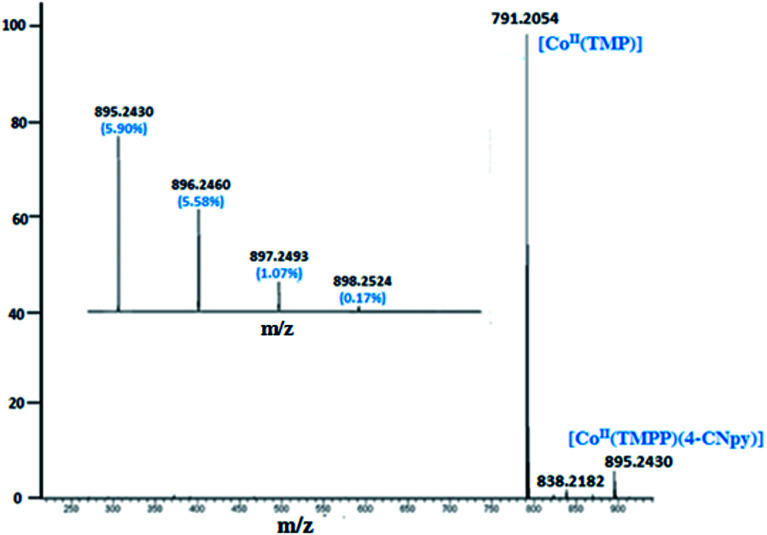
ESI-HRMS full spectrum of complex 1 showing the isotopic ratio. The solvent used is the dichloromethane with a concentration of 5 × 10^−5^ M.

**Fig. 2:**
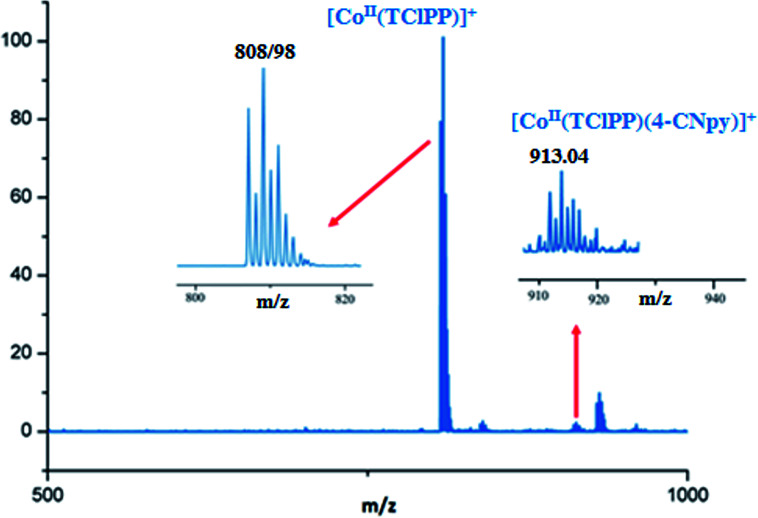
ESI-HRMS full spectrum of complex 2. The insets show enlarged views. The solvent used is the THF with a concentration of 5 × 10^−3^ M and diluted in methanol with a concentration of 5 × 10^−5^ M.

The ESI-mass studies in positive ion mode of complexes 1–2 have been carried out primarily to confirm the coordination of the 4-cyanopyridine to the Co(ii) cation of the [Co^II^(Porph)] (Porph = TMPP or TClPP) in solution. The ESI-HRMS data in dichloromethane solution of complex 1 does confirm the existence of the free axial ligand fragment [Co^II^(TMPP)]^+^ and the pentacoordinated [Co^II^(TMPP)(4-CNpy)]^+^ ion complex with experimental *m*/*z* values of 791.2054 and 895.2430, respectively. The HRMS spectrum of 1 along with the isotopic ratio is shown in Fig. 1. The ESI mass spectral peaks of [Co^II^(TClPP)(4-CNpy)] (2) are displayed in Fig. 2 where both coordinated and non-coordinated 4-CNpy cobaltous TClPP fragments [Co^II^(TClPP)]^+^ and [Co^II^(TClPP)(4-CNpy)]^+^ are shown with *m*/*z* values of 808.98 and 913.04, respectively. The corresponding theoretical *m*/*z* values of these two fragments are 808.45 and 913.55, respectively. Representative experimental and simulated isotopic pattern of 2 in the *m*/*z* 913 region corresponding to the [Co^II^(TClPP)(4-CNpy)]^+^ fragment is represented in Fig. SI-1.†

### 3.2. IR and proton NMR spectroscopic investigations

Complexes 1–2 exhibit characteristic IR spectra of the *meso*-porphyrin moieties and confirm the presence of the 4-CNpy axial ligand. The *ν*(CH) stretching frequency values of the TMPP and TClPP porphyrinato moieties and the 4-CNpy axial ligand of 1–2 are in the 3065–2830 cm^−1^ range, while the *δ*(CCH) bending frequency values of these two species are ∼1000 cm^−1^. The coordination of the 4-CNpy to the cobalt in complexes 1–2 is confirmed by the absorption bands at 2237 and 2234 cm^−1^ respectively corresponding to the *ν*(C≡N) stretching frequency which are slightly below that of the free 4-cyanopyridine compound (2243 cm^−1^) indicating that this axial ligand is coordinated to the Co(ii) center metal through the pyridyl group and not the nitrile moiety.^5^

The proton NMR spectroscopy is a very convenient technique to determine if a cobalt metalloporphyrin is a diamagnetic cobalt(iii) complex or a paramagnetic cobalt(ii) coordination compound with the 3d^6^ and 3d^7^ ground state electronic configurations of the Co(iii) and the Co(ii) cations, respectively.^20^ For cobalt(iii) *meso*-porphyrin derivatives, the β-pyrrolic and the phenyl ring protons are slightly shifted compared to those of the corresponding free base porphyrins with a chemical shift values in the range [8.5; 9] ppm and between 8.5 and 7.5 ppm for the phenyl protons (Table 1). However, the cobalt(ii) *meso*-arylporphyrins present ^1^H NMR spectra more downfield shifted, with chemical shift values of the β-pyrrole protons between 12 and 16.5 ppm and *δ* values of the phenyl protons in the range [13; 8.5] ppm (Table 1). Shirazi *et al.*,^33^ reported that the *δ* values of the *meso*-arylporphyrin protons are much higher for tetracoordinated Co(ii) porphyrin complexes than those for five and six-coordinated cobaltous metalloporphyrins (Table 1).

**Table 1:** Chemical shift values (in ppm) for 1–2 and selected free bases *meso*-arylporphyrins and cobalt *meso*-arylporphyrin complexes from ^1^H NMR spectra. The solvent used is the CDCl_3_

Compound	Hβ-pyrrolic protons	H-phenyl protons	H–OCH_3_^a^	Ref.
**Meso-porphyrins**				
H_2_TMPP	8.86	8.08; 7.27	4.10	This work
H_2_ClTPP	8.89	8.18; 7.74	—	This work
H_2_TpivPP^c^	8.82	8.70; 7.88; 7.50	—	34
H_2_TPP^b^	8.84	8.23; 7.91; 7.67; 7.26	—	34

**Cobalt(** **iii** **)-*meso*-porphyrin complexes**				
[Co^III^(TPP)Cl(DMI)]^b^^,^^d^	8.83	7.87; 7.65	—	35
[Co^III^(TPP)(DMI)]^+^^b^^,^^d^	8.95	7.86; 7.71	—	35
[Co^III^(TPP)(N_3_)(py)]^b^	9.22	8.38; 7.80	—	36
[Co^III^(TPP)Cl(py)]^b^	9.00	8.80; 7.70	—	36

**Cobalt(** **ii** **)-*meso*-porphyrin complexes**				
[Co^II^(TPP)]^b^	15.75	13.10; 9.80; 7.95	—	2
[Co^II^(TpivPP)]^c^	15.30	11.50; 10.90; 7.80	—	35
[Co^II^(TMPP)]	15.90	13.10; 9.43	5.25	This work
[Co^II^(TClPP)]	15.75	12.93; 9.9	—	This work
[Co^II^(TPP)(py)]^b^	12.50	8.5; 8.33; 7.82	—	37
[Co^II^(TPP)(HIm)]^b^^,^^e^	12.8	8.8; 8.40; 7.69	—	37
[Co^II^(TMPP)(4-CNpy)] CHCl_3_ (1)	14.64	11.42; 8.87; 9.01	5.01	This work
[Co^II^(TClPP)(4-CNpy)] (2)	14.61	11.53; 9.01; 9.42		This work

a H–OCH_3_ = protons of the OCH_3_ group in the *para*-phenyl positions of the H_2_TMPP porphyrin.

b TPP = *meso*-tetraphenylporphyrinato.

c TpivPP = *meso*-(α,α,α,α-tetrakis(*o*-pivalamidophenyl)porphyrinato).

d DMI = *N*,*N*′-dimethylimidazolylidene.

e HIm = imidazole.

The proton NMR spectra of our synthetic complexes 1–2 are reported in Fig. SI-2 and SI-3† while the chemical shift values of the β-pyrrole and phenyl protons of these species and other related compounds are given in Table 1.

The β-pyrrole protons of 1–2 resonate at ∼14.60 ppm while the aryl protons of these two cobaltous complexes are in range [11.53; 7.98] ppm which confirm that both derivatives are cobaltous metalloporphyrins. The chemical shift values of the OCH_3_ protons at the *para*-phenyl positions of the TMPP are 4.10, 5.25 and 5.01 ppm for the H_2_TMPP, [Co^II^(TMPP)] and [Co^II^(TMPP)(4-CNpy)] species, respectively. The protons of the 4-CNpy axial ligand at the 2,6 and 3,5 positions resonate at 9.01 and 7.98 ppm, respectively, for the TMPP derivative (1) and 9.42 and 8.81 ppm, respectively, for the TClPP-Co(ii) species (2). These values are quite different from those of the free 4-cyanopyridine molecule with chemical shifts of 7.96 ppm and 8.75 ppm indicating the coordination of the 4-CNpy in complexes 1–2.

### 3.3. UV-visible spectroscopy

**Fig. 3:**
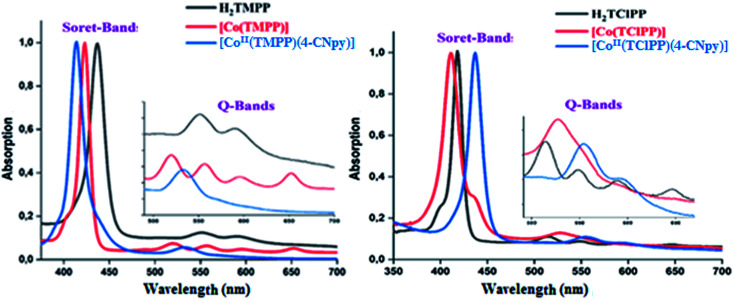
UV-visible spectra of H_2_TMPP, [Co^II^(TMPP)] and [Co^II^(TMPP)(4-CNpy)] (1) (left). And UV-visible spectra of H_2_TClPP, [Co^II^(TClPP)] and [Co^II^(TClPP)(4-CNpy)] (2) (right). The spectra were recorded in the dichloromethane with a concentration ∼10^−6^ M. The inset shows enlarged view.

The UV-visible spectra of the H_2_TMPP, H_2_TClPP free bases, the [Co^II^(TMPP)] and [Co^II^(TClPP)] starting materials and our cobaltous 4-cyanopyridine metalloporphyrins 1–2 are illustrated in Fig. 3 while Table 2 summarizes the UV-visible data of these species as well as for several related porphyrin species.

**Table 2:** UV-visible data of complexes 1–2 and a selection of *meso*-porphyrins and cobaltous metalloporphyrin coordination compounds. The solvent used is the dichloromethane (exceptions are indicated)

Compound	*λ*_max_ (nm) (*ε* × 10^−3^)					*E*_gap-opt_ (eV)	Ref.
	Soret band	*Q* bands					
**Free base *meso*-porphyrins**							
H_2_TClPP	417(320)	515(85)	548(36)	590(29)	646(26)	1.68	This work
H_2_TMPP	423(344)	521(24)	558(20)	597(16)	650(15)	1.76	This work
H_2_TTP^a^	420	616	552	594	640	1.86	38
H_2_TPBP	420(513)	516(17)	552(7)	591(5)	646(4)	1.82	5

**Cobaltous *meso*-porphyrin complexes**							
[Co^II^(TClPP)]	412(380)	529(64)	—	—		2.01	This work
[Co^II^(TMPP)]	414(450)	535(23)	—	—	—	2.11	This work
[Co^II^(TPP)]^b^	412	528	—	—	—		34
[Co^II^(TpivPP)]^c^	412	524	—	—	—		34
[Co^II^(TPBP)]^d^	412	528	—	—			7
[Co^II^(TMPP)(pip)]^e^	—	532	—	—	—		39
[Co^II^(TMPP)(py)]^f^	—	535	—	—	—		40
[Co^II^(TPBP)(4,4′-bpy)_2_]^d^^,^^e^	435(562)	552(30)	—	—	—		7
[Co^II^(TPP)(Hon)_2_]^b^^,^^g^	434	555	—	—			41
[Co^II^(TMPP)(4-CNpy)] (1)	437(420)	558(40)	600(35)	—	—	2.003	This work
[Co^II^(TClPP)(4-CNpy)] (2)	436(453)	556(49)	598(44)	—	—	1.971	This work

a H_2_TTP = *meso*-tetratolylporphyrin.

b TPP = *meso*-tetraphenylporphyrinato.

c TpivPP = α,α,α,α-tetrakis(*o*-pivalamidophenyl)porphyrinato.

d TPBP = *meso*-{tetrakis-[4-(benzoyloxy)phenyl]porphyrinato}.

e pip = piperidine.

f Spectrum recorded in toluene.

g Hon = 2-aminophenol.

In solution and in absence of any ligand (or coordinating solvent), cobalt(ii) metalloporphyrins are tetracoordinated species type [Co^II^(Porph)]. The addition of axial ligands lead to pentacoordinated or hexacoordinated derivatives type [Co^II^(Porph)(L)_*x*_]^−*m*^ (*m* = 0 and −1 for neutral and anionic L ligand respectively, *x* = 1 or 2). Staring material [Co^II^(Porph)] complexes present Soret band values at ∼412 nm. The addition of a N-donor neutral axial ligands leads to the redshift of the Soret band to ∼436 nm. In solid state, our two cobaltous-4-CNpy derivatives 1–2 are pentacoordinated as shown by X-ray molecular structures (see X-ray crystallographic section). In dichloromethane solution, 1–2 present very close UV-visible Soret and *Q* band values with Soret *λ*_max_ values of 437 and 436, respectively and *Q*(0,0) bands *λ*_max_ values of 558 nm and 556 nm, respectively. These values are very close to those of the reported hexacoordinated cobalt(ii) porphyrin complexes (Table 2) such as [Co^II^(TPP)(Hon)_2_] (Hon = 2-aminophenol).^41^ Unfortunately, for the known pentacoordinated Co(ii) porphyrin derivatives, only the *Q*(0,0) values are reported; *i.e.*, the *λ*_max_ of [Co^II^(TMPP)(pip)] (pip = piperidine) is 532 nm which is quite different from those of hexacoordinated cobaltous metalloporphyrins with a *λ*_max_ value ∼550 nm.^39^ Thus, the UV-visible investigation indicates that in solution, complexes 1–2 are pentacoordinated cobaltous metalloporphyrins. Since the UV-visible data of 1–2 are very close, the donor–acceptor character of the MeO and chlorine groups in the *para*-position of the phenyls of the TMPP and TClPP moieties have no effect on the electronic spectra of these two Co(ii) metalloporphyrins.

The porphyrins and the metalloporphyrins are known to present optical gap energy values (*E*_g-opt_) close to 2.00 eV.^5^ This is also the case for our synthetic free base porphyrins, the starting material [Co^II^(Porph)] complexes (Porph = TMPP or TClPP) and 1–2 (Table 2).

### 3.4. Fluorescence spectroscopy

Porphyrins and metalloporphyrins present two types of emission transitions: (1) the S_2_ S_0_ fluorescence of the B band (known as the Soret band) between the second excited singlet state S_2_ to the ground state S_0_, (2) the S_1_ S_0_ fluorescence of the *Q* bands between the first excited singlet state S_1_ to the ground state S_0_. In this case, we practically observe the two transitions: S_1_ [*Q*(0,0)] → S_0_ and S_1_ [*Q* (0,1)] → S_0_. Notably, the very weak S_2_ S_0_ fluorescence compared to those of the S_1_ S_0_ transitions and usually for porphyrin compounds, we are only interested to the former transition. Therefore, only the S_1_ S_0_ fluorescence is considered in the present investigation.

Guo *et al.*,^42^ reported that the insertion of Co(ii) in the porphyrin macrocycle has practically no effect on the positions of the *Q*(0,0) and *Q*(0,1) bands but the intensities of the bands of cobaltous metalloporphyrins are much weaker than those of the correspondent free base porphyrin. This is explained by the fact that cobaltous metalloporphyrins are paramagnetic low-spin (*S* = 1/2) complexes with the 3d^7^ ground state electronic configuration which enhance the intersystem crossing to the triplet state, thereby lowering the intensity of the fluorescence.

**Fig. 4:**
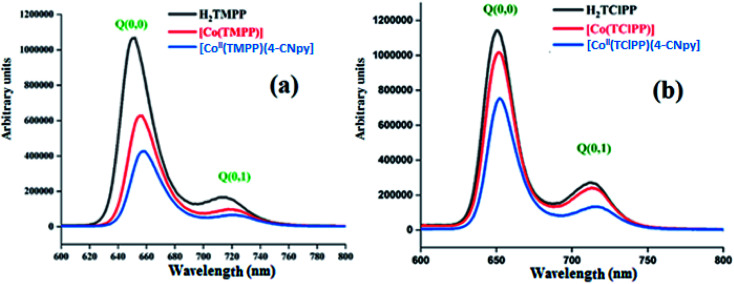
The emission spectra of (a) H_2_TMPP, [Co^II^(TMPP)] and [Co^II^(TMPP)(4-CNpy)] (1). (b) The emission spectra of H_2_TClPP, [Co^II^(TClPP)] and [Co^II^(TClPP)(4-CNpy)] (2). The spectra were recorded in dichloromethane solvent with a concentration ∼10^−6^ M. The excitation wavelength values are given in Table 3.

The S_1_ → S_0_ fluorescence spectra of H_2_TMPP, [Co^II^(TMPP)] and [Co^II^(TMPP)(4-CNpy)] (1) are shown in Fig. 4a, while those of H_2_TClPP, [Co^II^(TClPP)] and [Co^II^(TClPP)(4-CNpy)] (2) are shown in Fig. 4b. Representative fluorescence decays for 1–2 are shown in Fig. SI-4† and the fluorescence data of these species and those of several *meso*-porphyrins and cobalt(ii) metalloporphyrins are given in Table 3.

**Table 3:** Emission parameter values of several *meso*-porphyrins and a selection of cobalt(ii) *meso*-metalloporphyrins

Compound	*λ*_exci_ a	*Q*(0,0)	*Q*(0,1)	*ϕ*_f_ b	*τ*_f_ c (in ns)	Solvent	Ref.
**Free base *meso*-porphyrins**							
H_2_TMPP	424	656	719	0.082	7.16	CH_2_Cl_2_	This work
H_2_TClPP	418	651	714	0.089	7.42	CH_2_Cl_2_	This work
H_2_TPP^d^	—	654	712	0.11	—	CH_2_Cl_2_	43 and 44
H_2_TPP^d^	—	656	717	0.09	—	CH_2_Cl_2_	45
H_2_TPP^d^	—	653	722	0.12	9.6	DMF	46

**Tetracoordinated cobalt(** **ii** **) *meso*-metalloporhyrins**							
[Co^II^(TPBP)]^e^	552	653	719	0.032	—	CH_2_Cl_2_	7
[Co^II^(TMPP)]	414	655	719	0.035	6.02	CH_2_Cl_2_	This work
[Co^II^(TClPP)]	412	652	713	0.04	6.10	CH_2_Cl_2_	This work

**Pentacoordinated and hexacoordinated cobalt(** **ii** **) *meso*-porphyrins**							
[Co^II^(TPBP)(4,4′-bipy)_2_]^e^^,^^f^	550	652	718	0.036	—	CH_2_Cl_2_	7
[Co^II^(TMPP)(4-CNpy)]·CHCl_3_ (1)	437	652	717	0.054	1.97	CH_2_Cl_2_	This work
[Co^II^(TClPP)(4-CNpy)] (2)	439	653	714	0.06	1.997	CH_2_Cl_2_	This work

a *λ*_exci_ = wavelength value of excitation.

b *ϕ*_f_ = fluorescence quantum yields.

c *τ*_f_ = fluorescent lifetime.

d TPP = *meso*-tetraphenylporphyrinato.

e TPBP = *meso*-{tetrakis-[4-(benzoyloxy)phenyl]porphyrinato}.

f 4,4′-bipy = 4,4′-bipyridine.

These two figures and Table 3 show that the *λ*_max_ values of the *Q*(0,0) and *Q*(0,1) bands of H_2_TMPP, H_2_TClPP, the corresponding cobaltous porphyrin species and the coordinated 4-CNpy [Co^II^(Porph)(4-CNpy)] (Porph = TMPP and TClPP) species are very close (∼652 nm and ∼715 nm, respectively). Our previous studies have shown that following the insertion of the cobalt in the porphyrin core, the positions of the *Q* bands are practically the same while the intensities of the cobaltous metalloporphyrins are much weaker than those of the H_2_TMPP and H_2_TClPP free bases.^42^ For the two *meso*-porphyrins free bases, the fluorescence quantum yields (*ϕ*_f_) values are 0.082 and 0.089, respectively, while those of the corresponding [Co^II^(TMPP)] and [Co^II^(TClPP)] starting materials are 0.035 and 0.04, respectively. The coordination of the 4-CNpy leads to a slight increase of the quantum yield compared to those of the tetracoordinated complexes [Co^II^(Porph)] (Porph = TMPP and TClPP) with *ϕ*_f_ values of 0.054 and 0.06 for 1–2, respectively. The *λ*_max_ values of the *Q*(0,0) and *Q*(0,1) bands and the *ϕ*_f_ values of our two cobaltous derivatives are very close to those of the related [Co^II^(TPBP)(4,4′-bipy)] (TPBP = *meso*-tetra[4-(benzoyloxy)phenyl]porphyrinato) compound^7^ (Table 3). The fluorescent lifetime (*τ*_f_) values of our synthetic cobalt(ii) metalloporphyrins are smaller than those of the free *meso*-porphyrins H_2_TMPP and H_2_TClPP, as expected (Table 3).

### 3.5. X-ray structures of complexes 1–2

Complex 1 crystallizes in the monoclinic crystal system with *C*2/*c* space group, while complex 2 crystallizes in the triclinic crystal system with *P*1̄ space group. One [Co^II^(TMPP)(4-CNpy)] molecule was found in the asymmetric unit of 1 with one CHCl_3_ solvent molecule. In the case of complex 2, the symmetric unit is made of one [Co^II^(TClPP)(4-CNpy)] molecule and one very disordered dichloromethane solvent molecule which was eliminated using the SQUEEZE procedure of the PLATON program.^29^

**Fig. 5:**
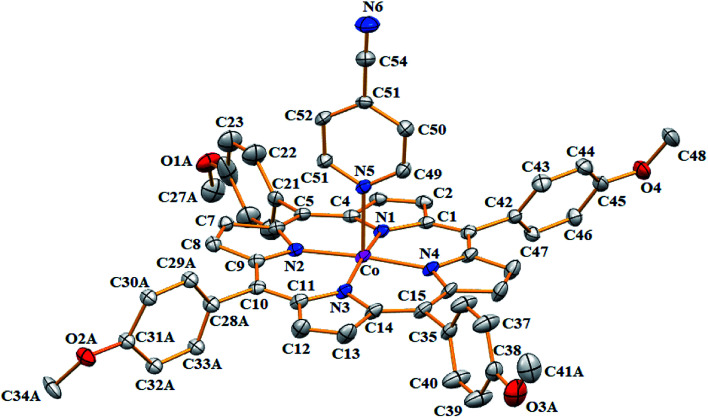
ORTEP diagram of [Co^II^(TMPP)(4-CNpy)] (1). Only the major disorder fragments are shown and the hydrogen atoms are not represented for clarity. The ellipsoids are drawn at 40%.

**Fig. 6:**
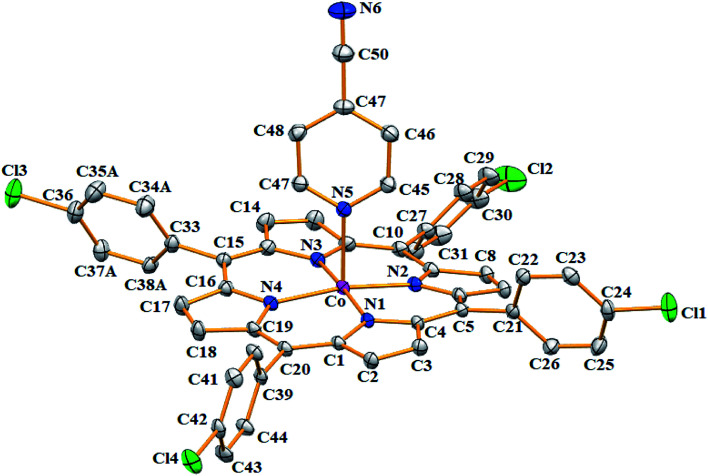
ORTEP diagram of [Co^II^(TClPP)(4-CNpy)] (2). Only the major disorder fragments are shown and the hydrogen atoms are not represented for clarity. The ellipsoids are drawn at 40%.

The ORTEP drawings of these two cobaltous metalloporphyrins 1–2 are illustrated in Fig. 5 and 6, respectively. For both complexes 1–2, the cobalt(ii) central ion are chelated by the four pyrrole N atoms of the porphyrinate anion and the 4-CNpy axial ligand occupies the apical site of the distorted square-pyramidal coordination polyhedral (Fig. SI-5†). The 4-CNpy is coordinated to the Co(ii) center ion through the N-pyridyl cycle as is the case for all known 4-cyanopyridine metalloporphyrins. Fig. SI-5† represents the coordination polyhedral of the cobaltous center ion of the two 4-cyanopyridine porphyrin derivatives 1–2 which shows that the angles and distances of both two “Co-4-CNpy–porphyrin” systems are very close. Nevertheless, the Co-N(4-CNpy) value for the TClPP derivative (2) [2.196(3) Å] is smaller than that of the TMPP species (1) [2.209(3) Å]. These distances are very close to the [2.159–2.202 Å] range of the related Co(ii)-4-CNpy non-porphyrinic complexes (Table 4). For 1–2, as expected, the C–C–N group of the 4-CNpy ligand is linear where the C51–C54–N6 and C47–C50–N6 angles values are 178.7(5)° and 179.1(5)°, respectively. The C–C and C≡N distances values of the same C–C–N moiety are 1.449(5) Å and 1.139(5) Å, respectively, for complex 1 and 1.446(5) Å and 1.146(5) for complex 2, respectively.

**Table 4:** Selected bond lengths (Å) and angles (°) for [Co^II^(TMPP)(4-CNpy)]·CHCl_3_ (1), [Co^II^(TClPP)(4-CNpy)] (2) and several related porphyrinic and non-porphyrinic complexes

Complex	Porphyrin core deformation type^a^	M–N_p_^b^	M–X_L_^c^	M–P_C_^d^	Ref.
**Co(** **ii** **) porphyrin complexes**					
[Co^II^(TPP)]^e^	+++Ruf	1.923	—	0.050	48
[Co^II^(TPP)]^e^	+++Ruf	1.949	—	0.009	51
[Co^II^(TPP)(NO_2_)(Lut)]^−^e^,^^f^	++Ruf	1.959(2)	2.017(2)(Lut), 1.925(2) (NO_2_)	0.044	49
[Co^II^(TPP)(1-MeIm)] e^,^^g^	−Ruf	1.978(3)	2.157(3)	0.139	52
[Co^II^(TPP)(pip)_2_]^e^^,^^h^	Planar	1.987	2.436(2)	0.000	50
[Co^II^(TCPP)(py)_2_]^i^	++Ruf	1.961	1.958	0.000	53
[Co^II^(OEP)(DMAP)]^j^^,^^k^	Planar	1.981(3)	2.191(2)	0.156	54
[Co^II^(TPP)(pipz-S)]^l^	++−Sad, −Ruf	1.989(5)	2.241(5)	0.134	55
[Co^II^(TMPP)(4-CNpy)]·CHCl_3_ (1)	Ruf, +sad	1.984(3)	2.209(3)	0.1404(8)	This work
[Co^II^(TClPP)(4-CNpy)] (2)	++Ruf, +sad	1.977(3)	2.196(3)	0.1440(7)	This work

**4-Cyanopyridine metalloporphyrins**					
[Ni^II^(PFPP)(4-CNpy)_2_]^m^	Planar	2.283	2.220	—	56
[Fe^III^(TPP)(4-CNpy)_2_]^e^	+++Ruf, sad	1.952(4)	1.996/2.008	—	21
[Fe^II^(TMP)(4-CNpy)_2_]^n^	Planar	1.992	1.996	—	22
[{Rh^III^(OEP)}_2_(μ_2_-4-CNpy)]^j^	Planar	2.032(4)	2.273(4)	—	22
[Zn^II^(TBAP)(4-CNpy)]^o^	+Ruf, +sad	2.060(6)	2.159(2)	—	57

**4-Cyanopyridine-Co(** **ii** **) non-porphyrinic complexes**					
[Co^II^(SO_4_)(4-CNpy)_2_(H_2_O)_3_]	—	—	2.135(1)/2.200(1)	—	58
[Co^II^(L1)_2_(4-CNpy)_2_]^p^	—	—	2.159	—	59
{[Co^II^(μ-Br)_2_(4-CNpy)_2_]}_*n*_	—	—	2.202	—	60

a See the description of different types of the porphyrin core deformation in the text, planar designate a planar porphyrin core. +: moderate, ++: important, +++: very important and “−”: weak deformation.

b M–N_p_ = average equatorial distance between the center metal and the nitrogen atoms of the pyrroles.

c M–X_L_ = distance between the metal atom and the coordinated atoms of the axial ligands.

d M–P_C_ = distance between the metal atom and the mean plane made by the 24-atom core of the porphyrin (P_C_).

e TPP = *meso*-tetraphenylporphyrinato.

f Lut = 2,6-lutidine.

g 1-MeIm = 1-methylimidazole.

h pip = piperidine.

i TCPP = *meso*-tetra(4-carboxyphenyl)porphyrinato.

j OEP = octaethylporphyrin.

k DMAP = 4-(dimethylamino)pyridine.

l pipz-S = (piperazin-1-yl)sulfonyl)naphthalen-1-amine.

m PFPP = *meso*-tetra(pentafluorophenyl)porphyrinato.

n TMP = *meso*-tetramesitylporphyrinato.

o TPBP = *meso*-{tetrakis-[4-(benzoyloxy)phenyl]porphyrinato}.

p L1 = 3,5-di-*t*-butylbenzosemiquinonato.

For the case of the Co-TMPP derivative (1), the projection of the 4-CNpy plane is very close to the *trans* N_p_–Co–N_p_ vector with an angle (*φ*) of 3.0(2)° while for the Co-TClPP species (2), the value of dihedral angle between the projection of the 4-CNpy and the closest *cis* N_p_–Co–N_p_ angle is 20.5(2)°. It should be noted that for hemoproteins and the majority of metalloporphyrins, the planar axial ligand (type imidazole or pyridine) is located parallel to the *trans meso*-positions with a *φ* dihedral angle value of about 45°. For our TMPP-4-CNpy cobaltous derivative (1) the very short *φ* angle value could be explained by the intermolecular interaction between the nitrogen N6 of the 4-CNpy ligand and the hydrogen H17 of a pyrrole group of an adjacent [Co^II^(TMPP)(4-CNpy)] molecule (see the crystal packing description below).

**Fig. 7:**
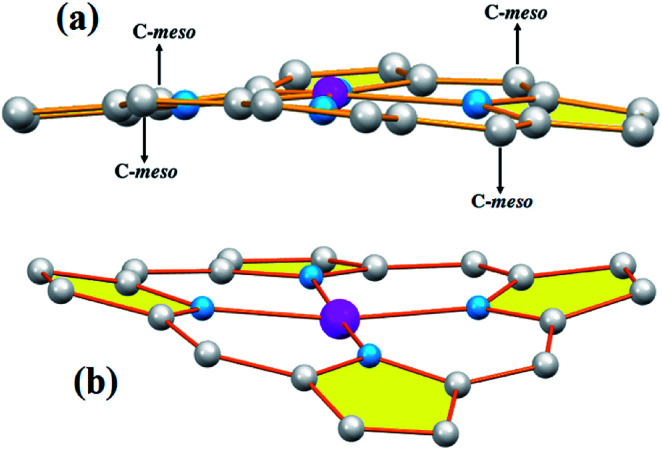
Schematic representation of the porphyrin core ruffling (ruf) deformation (a) and the saddle (sad) deformation (b).

**Fig. 8:**
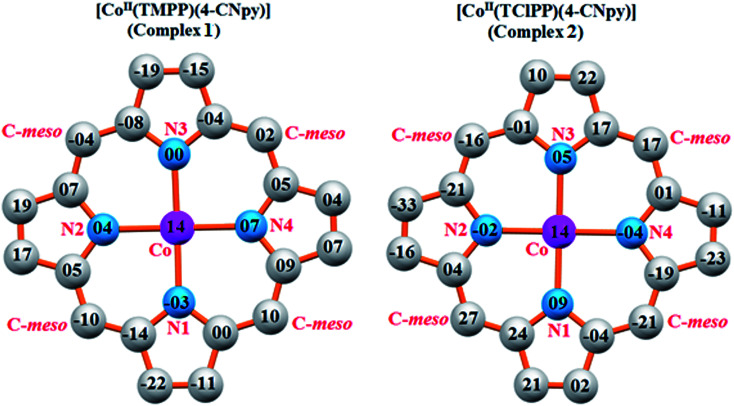
Formal diagrams of the porphyrinato cores of 1 (left) and 2 (right). The displacement of each atom from the mean plane of the 24-atom porphyrin macrocycle in given in units of 0.01 Å.

Iimura *et al.*,^47^ reported that the value of the mean equatorial distance between the cobalt cation and the four nitrogen atoms of the porphyrin macrocycle (Co–N_p_) is directly related to the ruffling deformation of the porphyrin core (Fig. 7) where the Co–N_p_ bond length decreases as the ruffling of the porphyrin cycle increases. The ruffling deformation of the porphyrin core is indicated by the high values of the displacement of the *meso*-carbon atoms above and below the porphyrin mean plane. The shortest Co–N_p_ distance (1.923 Å) reported in Table 4, corresponds to the [Co(TPP)] complex^48^ for which the ruffling is the most important among all the known Co(ii) porphyrins. The correlation between the Co–N_p_ distance and the ruffling deformation is also observed for the pentacoordinated cobaltous metalloporphyrins (Table 4). For instance, the Co–N_p_ bond length of [Co^II^(TPP)(NO_2_)(lut)] (lut: 2,2-luthidine)^49^ is 1.959(2) Å while for [Co^II^(TPP)(pip)_2_]^50^ (pip = piperidine), the Co–N_p_ distance is 1.987 Å. Thus, the former complex exhibits high ruffling deformation while the later one presents a planar porphyrin core. For our two Co(ii) derivatives 1–2, the displacements of each atom from the 24-atom core plane in units of 0.01 Å are given in Fig. 8. It has been reported that high ruffling of the porphyrin core could originate from strong π-acid axial ligands such as 4-cyanopyridine^24^ which can explain the short Co–N_p_ distance for the TClPP derivative (2) with a value of 2.977(2) Å. Nevertheless, for the TMPP-4-CNpy derivative (1), the Co–N_p_ distance is quite high with a value of 2.985(2) Å. This could be because in the TMPP porphyrinate, the donor effect of the methoxy group in the *para*-positions of the porphyrin phenyls (which is more important than those of the chlorine atoms in the *para*-positions of the TClPP moiety) destabilizes the π orbitals of the porphyrin core.^21^ It is noteworthy, that the Co–N_p_ bond length values of 1–2 are not affected by the saddling deformation of the porphyrin macrocycle (Table 4).

A part of the crystal packing of the [Co^II^(TMPP)(4-CNpy)] complex (1) is illustrated by Fig. SI-6.† The [Co^II^(TMPP)(4-CNpy)] molecules are located up-side-down in the lattice where the chloroform solvent molecules are located in channels parallel to the [010] direction. The C52 aromatic carbon of the 4-CNpy axial ligand of one [Co^II^(TMPP)(4-CNpy)] molecule is hydrogen bonded to the nitrogen N6 of the 4-CNpy axial ligand of a nearby up-side-down [Co^II^(TMPP)(4-CNpy)] molecule with a C52–H52⋯N6 distance of 3.553(6) Å. The crystal lattice of complex 1 is also sustained by weak C–H⋯O (oxygen of the OMe group in the *para* position of the TMPP) and C–H⋯Cg where Cg is the centroid of a TMPP phenyl ring (Tables SI-3, SI-4 and Fig. SI-6†). Additionally, the chloroform solvent molecule is involved in weak intermolecular interactions of type C–H⋯Cl between a phenyl carbon of one TMPP porphyrinato and one chlorine atom of the CHCl_3_ solvent molecule (Tables SI-2, SI-3 and Fig. SI-6).†

A three-dimensional non-conventional hydrogen-bonded network is formed in the crystal lattice of the [Co^II^(TClPP)(4-CNpy)] (2) by C–H⋯N and C–H⋯Cg where Cg is the centroid of a pyrrole ring of the TClPP moiety. Indeed, the carbon C48 of one [Co^II^(TClPP)(4-CNPy)] molecule is weakly H-bonded to the nitrogen N6 of the CN moiety of the 4-CNpy on an up-side-down neighboring [Co^II^(TClPP)(4-CNpy)] with a C48–H48⋯N6 distance of 3.527(6) Å (Fig. SI-7, Table SI-3 and SI-4†). The carbon C23 of a phenyl ring of one TClPP-Co(ii) derivative is linked to the centroid Cg1 of the N1/C1–C4 pyrrole ring of a nearby [Co^II^(TClPP)(4-CNpy)] with a C23–H23⋯Cg1 distance of 3.613(4) Å. The crystal packing of 2 is further consolidated by π–π stacking Cg1⋯Cg2 interactions where Cg2 is the centroid of the N2/C6–C9 pyrrole ring (Fig. SI-7†).

**Fig. 9:**
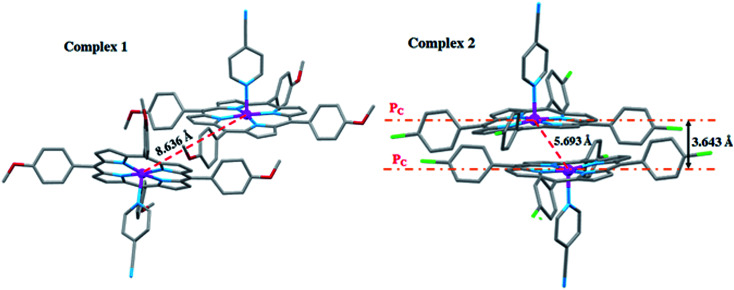
Drawing showing the Co⋯Co interaction for complexes 1–2. P_C_ is the 24-atom porphyrin core.

The [Co^II^(TClPP)(4-CNpy)] complex (2) exhibits short Co⋯Co distance with a value of 5.663 Å with a distance value between two adjacent 24-atom mean plan porphyrin rings (P_C_) of 3.643 Å leading to a supramolecular dimer (Fig. 9). However, this is not the case for [Co(TMPP)(4-CNpy)] (1) with a much longer Co⋯Co distance value of 8.636 Å.

The β-substituted octaethylporphyrin (H_2_OEP) complexes in solid state can also form supramolecular dimers known as π–π dimers.^61^ The stability of these species is consolidated by π interaction between the two 24-atom plans (porphyrin cores) of one supramolecular dimer, *e.g.* in the case of the [Fe^III^(OEP)(O_2_C_2_Cl_3_)]·CHCl_3_ (ref. 62) species, the Fe⋯Fe distance is 5.45 Å. Although the *meso*-arylporphyrin complexes were originally synthesized to prevent porphyrin aggregation, the phenyl groups are not sufficiently sterically bulky to completely prevent the formation of supramolecular dimers. Thus, as reported by Scheidt and Lee,^63^ there are a number of “dimeric” TPP (*meso*-tetraphenylporphyrinato) metalloporphyrins observed in the solid state. In 2017, Nasri *et al.*,^64^ reported the molecular structures of two *meso*-arylporphyrin manganese(iii) complexes: [Mn^III^(TPP)(TBA)] (TPP = *meso*-tetraphenylporphyrinato and TBA = trichloroacetato axial ligand) and [Mn^III^(TBrPP)(TCA)] (TBrPP = *meso*-tetra-(*para*-bromophenylporphyrinato)). The Mn⋯Mn distance values are 5.57 Å and 4.80 Å for the TPP and TBrPP derivatives, respectively. For these two species, the smaller Mn⋯Mn distance values, in the case of the TBrPP derivative, are related to its smaller deformation of the porphyrin core leading to a close contact between the two center ions.

For our two 4-CNpy derivatives 1–2, the high value of the Co⋯Co distance in the case of the [Co^II^(TMPP)(4-CNpy)] complex (1), which is 8.636 Å, could be related to the bulky methoxy groups of the TMPP porphyrinato which is not the case of the [Co^II^(TClPP)(4-CNpy)] species (2). This coordination compound presents a much smaller Co⋯Co distance of 5.693 Å, therefore enabling the formation of a supramolecular dimer. Additionally, the electron withdrawing effect of the Cl compared to the OMe causes a more efficient π–π stacking and results in a shorter Co⋯Co distance.

The π–π supramolecular dimer in the case of (2) is stabilized, as mentioned above, by C–H⋯Cg intermolecular interactions (Cg is the centroid of a pyrrole ring) and also by the Cg1⋯Cg2 π–π interactions where Cg1 and Cg2 are the centroids of pyrrole rings of two adjacent [Co^II^(TClPP)(4-CNpy)] molecules forming the supramolecular dimer (Fig. SI-7†).

### 3.6. Hirshfeld surface analysis

**Fig. 10:**
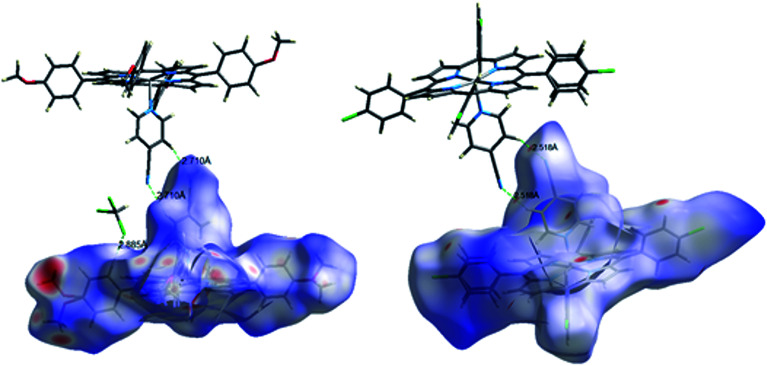
Molecular Hirshfeld surfaces mapped with *d*_norm_ about a reference molecule of complexes 1 (left) and complex 2 (right) highlighting the intermolecular hydrogen bonds in the crystal packing of 1–2.

Hirshfeld surface serves as a powerful tool for gaining additional insight into the intermolecular interactions^65^ in addition to the classic results given by the PLATON program which were just discussed in the previous crystallographic section. The Hirshfeld surfaces of our two synthetic Co(ii)-4-CNpy species 1–2, which are shown as transparent to allow visualization of the asymmetric unit of the given crystal structure, are illustrated in Fig. 10. These Hirshfeld surfaces have been mapped over a “*d*_norm_” range of −0.7287 to 1.5763 Å for 1 and from −0.2021 to 3.036 Å for 2. Fingerprint plots for 1–2 are represented in Fig. SI-8 and SI-9.† The red shaded areas on the Hirshfeld surface indicate the presence of close contacts and areas without close contacts are shown in blue color. For our Co-TMPP-4-CNpy derivative (1), 47% of the intermolecular interactions are related to the H⋯H contacts where the proportion of C⋯H/C⋯H, N⋯H/H⋯N, O⋯H/H⋯O and Cl⋯H/H⋯Cl are 24.8%, 8.2%, 8.7% and 5.3%, respectively.

In the case of the Co-TClPP-4-CNpy species (2), the H⋯H contacts represents 36.3% of the total intermolecular interactions with the following proportions: C⋯H/H⋯C of 17%, N⋯N/H⋯N of 8.5% and a very weak Cl⋯H/H⋯Cl of 22.6% between the chlorine atoms of one TClPP moiety and several phenyl carbons of an adjacent TClPP species.

### 3.7. Cyclic voltammetry

**Scheme 1:**
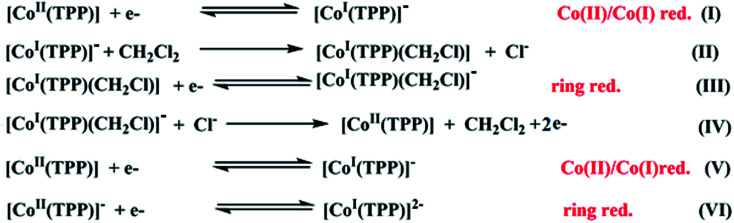
Electrochemical reduction reactions of [Co^II^(TPP)] in dichloromethane solvent.^62,63^

Several cyclic voltammetry (CV) investigations have been reported on cobaltous metalloporphyrins since 1975.^17^ For the [Co^II^(TPP)] coordination compounds, it has been shown that in nonchlorinated solvents such as DMSO and THF and in dichloromethane solvent, these derivatives exhibit three one-electron reversible oxidation waves where the first one corresponds to the Co(ii)/Co(iii) oxidation followed by two porphyrin core oxidations. The reduction of the [Co^II^(TPP)] compound in nonchlorinated solvents (Scheme 1) starts with the Co(ii)/Co(i) reduction (eqn (I)) then a one-electron porphyrin ring reduction. The electrogenerated Co(i) species [Co^I^(TPP)]^−^ is modestly stable in nonchlorinated solvents, but reacts instantly with the dichloromethane solvent to form a transient carbon-bonded species identified as the [Co^I^(TPP)(CH_2_Cl)] complex (eqn (II)) which will be reduced to the [Co^I^(TPP)(CH_2_Cl)]^−^ derivative (eqn (III)). The [Co^I^(TPP)]^−^ ion complex is regenerated successively by reactions (IV) and (V) then, an irreversible porphyrin core reduction leads to the [Co^I^(TPP)]^2−^ ion complex (eqn (VI)).^66^

The CV of the two starting materials [Co^II^(TMPP)] and [Co^II^(TClPP)] and complexes 1–2 were recorded in the non-coordinating dichloromethane solvent under argon using the tetra-*n*-butylammonium perchlorate (TBAP) as the supporting electrolyte (0.2 M) and all potential values are given in volt *versus* SCE. For [Co^II^(TClPP)] and [Co^II^(TMPP)] (Table 5), the values of the half potential first reversible Co(ii)/Co(i) reduction (eqn (I)) (MR1,MO1) are −0.88 and −0.70 V respectively. The anodic irreversible wave shown at −0.29 V for [Co^II^(TClPP)], which is not seen for [Co^II^(TMPP)], could be explained, as just mentioned above,^66^ by the reduction involving the [Co^I^(TClPP)(CH_2_Cl)] and [Co^I^(TClPP)(CH_2_Cl)]^−^ intermediates. The first porphyrin ring reduction (eqn (VI)) of the TClPP and TMPP cobaltous tetracoordinated species present *E*_1/2_[R4,O4] values of −1.40 and −1.36 V, respectively.

**Table 5:** Electrochemical data a for H_2_TMPP, H_2_TClPP, [Co^II^(TMPP)], [Co^II^(TClPP)], complexes 1–2 and a selection of *meso*-porphyrins and Co(ii) *meso*-metalloporphyrins. All data are obtained from voltammograms recorded in dichloromethane

	Oxidations				Reductions			Ref.
	1^st^ Porph oxid. (O1,R1)	2^nd^ Porph oxid. (O2,R2)	3^rd^ Porph oxid. (O3,R3)	Oxid. Co(ii)/Co(iii) (MO2)	1^st^ Porph red. (R4,O4)	2^nd^ Porph red. (R5,O5)	Red Co(ii)/Co(i) (MR1,MO1)	
	*E*_1/2_ b	*E*_1/2_	*E*_1/2_	*E*_1/2_	*E*_1/2_	*E*_1/2_	*E*_1/2_	
H_2_TPP	1.02	1.26	—	—	−1.20	−1.55	—	68
H_2_TPBP	0.95	1.36	1.48	—	−1.12	−1.53	—	5
H_2_TMPP	1.02	1.19	1.67^c^	—	−1.19	−1.52	—	This work
H_2_TClPP	1.00	1.23	1.53	—	−1.09	−1.41	—	This work
[Co^II^(TPP)]	1.16	—	—	0.98	−1.40^c^	—	−0.83	69
[Co^II^(TPP)]	0.97	—	—	0.78	—	—	−0.85	70
[Co^II^(TPP)]	0.91	—	—	0.75	—	—	—	17
[Co^II^(TClPP)]	1.00	1.26	1.85	0.60^c^	−1.40	—	−0.88	This work
[Co^II^(TMPP)]	0.93	1.20	—	0.70^c^	−1.36	—	−0.70	This work
[Co^II^(TPP)(py)_*x*_]	—	—	—	−0.12	—	—	−1.16	69
[Co^II^(TMPP)(4-CNpy)] (1)	0.89	1.25	1.78	0.47	−1.43	—	−0.94	This work
[Co^II^(TClPP)(4-CNpy)] (2)	1.13	1.31	—	0.42	−1.32	—	−0.92	This work

a The potentials are reported *versus* SCE.

b *E*_1/2_ = half wave potential.

c Irreversible wave.

**Fig. 11:**
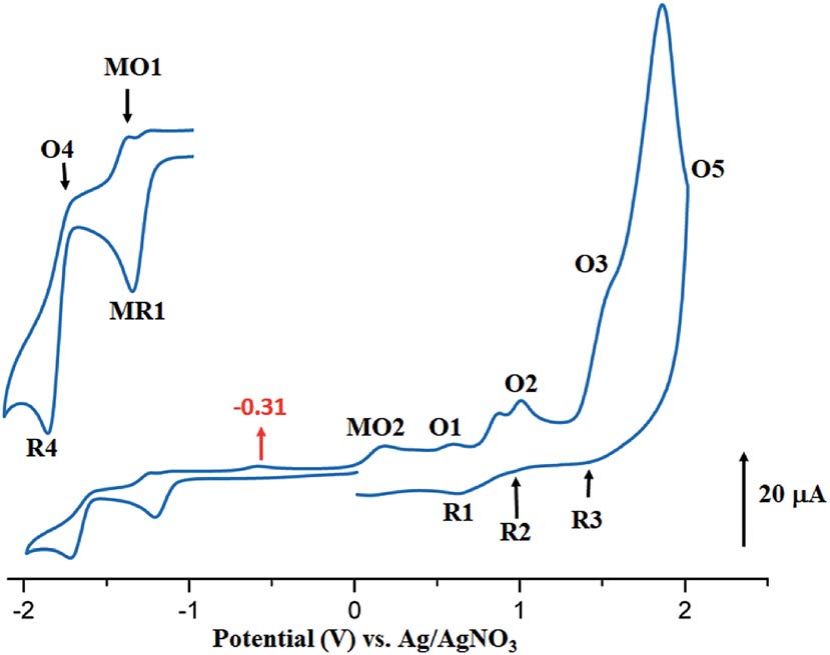
Cyclic voltammogram of 1. The solvent is CH_2_Cl_2_ and the concentration is *ca.* 10^−3^ M in 0.2 M TBAP, 100 mV s^−1^, vitreous carbon working electrode (*Ø* = 2 mm). The inset shows enlarged view.

**Fig. 12:**
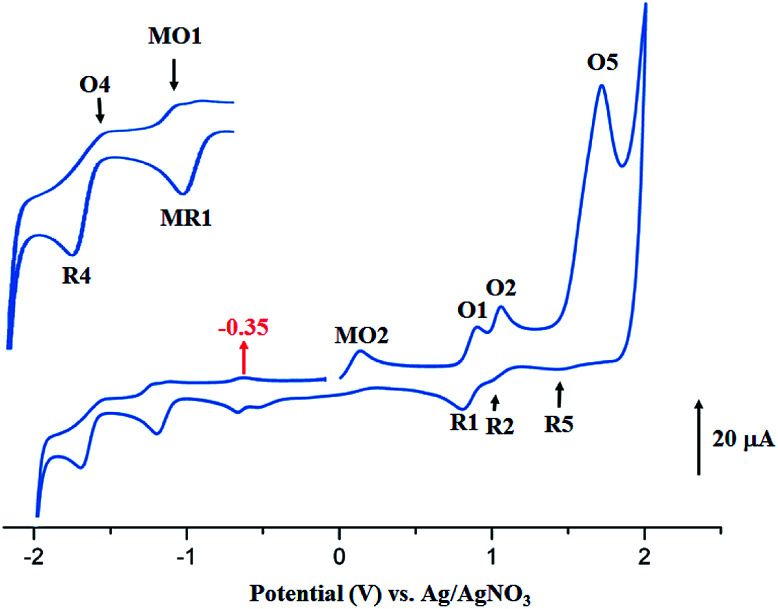
Cyclic voltammogram of 2. The solvent is CH_2_Cl_2_ and the concentration is *ca.* 10^−3^ M in 0.2 M TBAP, 100 mV s^−1^, vitreous carbon working electrode (*Ø* = 2 mm). The inset shows enlarged view.

The anodic region of the cyclic voltammograms of [Co^II^(TClPP)] and [Co^II^(TMPP)] (Table 5, the voltammograms of these species are not reported), presents: (1) an oxidation of the center ion [Co(ii)/Co(iii)] (MO2) with *E*_ap_ (*E*_ap_ = anodic peak potential) values of 0.60 for the TClPP derivative 0.65 V for the TMPP species, (2) a first oxidation of the porphyrin ring (O1,R1) with *E*_1/2_ values of 1.02 and 0.92 V for the TClPP and TMPP derivatives, respectively, (3) these two cobaltous complexes exhibit a reversible second ring oxidation waves (O2,R2) with *E*_1/2_ values of 0.26 and 1.20 V, respectively, (4) unlike [Co^II^(TMPP)], complex [Co^II^(TClPP)] presents a third irreversible oxidation of the porphyrin core (O3,R3) with *E*_1/2_ value of 1.85 V and (5) an irreversible oxidation wave (O5) for both two cobaltous tetracoordinated TClPP and TMPP complexes are shown which *E*_ap_ values ∼2.29 V for both species which can be attributed to the phenyl rings oxidation of the *meso*-porphyrin.^67^ For the [Co^II^(TMPP)] complex, the irreversibility of this oxidation is probably due to chemical reactions involving unstable electrogenerated radicals leading to an unidentified species reduced at about 0.18 V (R5). The cyclic voltammograms of complexes [Co^II^(TMPP)(4-CNpy)] and [Co^II^(TClPP)(4-CNpy)] (1–2) are illustrated in Fig. 11 and 12 and the CV data of these two cobaltous 4-CNpy derivatives are reported in Table 5 along with those of several reported porphyrinic species. The CV data of 1–2 are similar to those of the [Co^II^(TMPP)], [Co^II^(TClPP)] and other reported cobalt(ii) *meso*-porphyrins (Table 5). The first one-electron reversible reduction of 1–2 correspond to the Co(ii)/Co(i) center metal reduction (RM1,OM1) with *E*_1/2_ values of −0.94 and −0.92 V, respectively. Then a first ring reduction (R4,O4) occurs with *E*_1/2_ values of −1.35 and −1.32 V for 1–2, respectively. An irreversible wave with *E*_ap_ values of −0.31 and −0.35 V for 1–2 respectively, could be attributed to the reduction involving the [Co^I^(Porph)(CH_2_Cl)] and [Co^I^(TClPP)(CH_2_Cl)]^−^ (Porph = TClPP or TMPP) intermediates. A first irreversible oxidation wave of 1–2 corresponding to Co(ii)/Co(iii) oxidation (MO2,MR2) are shown with *E*_ap_ values of 0.47 and 0.42 V, respectively and a first reversible ring oxidation (O1,R1) of these derivatives present *E*_1/2_ values of 0.89 and 1.18 V, respectively. Then, a second reversible ring oxidation wave (O2,R2) occurs at *E*_1/2_ values of 1.25 and 1.31 V for 1–2 respectively. The [Co^II^(TMPP)(4-CNpy)] (1) and unlike the complex [Co^II^(TClPP)(4-CNpy)] (2) shows a quasi-reversible third ring oxidation wave (O3,R3) with *E*_1/2_ value of 1.78 V. Both cyclic voltammograms of complexes 1–2 present an irreversible oxidation wave with *E*_pa_ = 2.29 for 1 and 2.01 V for 2 attributed to the phenyl rings oxidation (O5) of the *meso*-porphyrins.

Thus, summarizing the CV investigation: (i) the values of the potential of the center metal Co(ii)/Co(i) reduction and especially the values of the potential of the center metal oxidation Co(ii)/Co(iii) of the cobaltous 4-CNpy derivatives (1–2) are shifted to more negative values compared to those of the starting material of tetracoordinated complexes [Co^II^(Porph)] (Porph = TMPP or TClPP), (ii) the values of oxidation and reduction potential do not depend on the nature of the *meso*-porphyrin (TMPP or TClPP porphyrinato ligands). Notably, the fact that the larger shift to more negative potential values observed for the Co(ii)/Co(iii) oxidation compared to those of the Co(ii)/Co(i) reduction suggests that Co(iii) complexes are stabilized to a greater degree than the cobalt(ii) metalloporphyrin complexes.^67^

### 3.8. Kinetic adsorption and degradation of the methylene blue dye using complexes 1–2

**Fig. 13:**
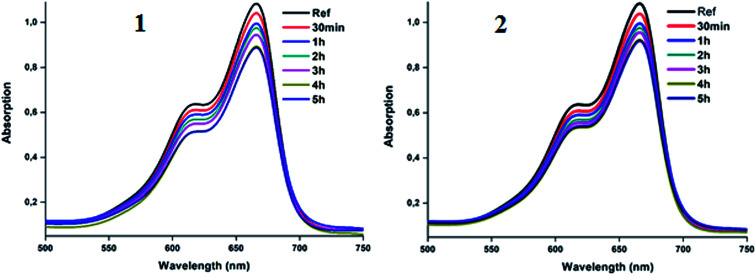
Variation of the *λ*_max_ values of the absorption bands of MB dye in aqueous solution in the presence of complex 1 (10 mg) (left) and complex 2 (10 mg) (right). The concentration of MB is 10 mg l^−1^ and pH = 6.

The utilization of our synthetic Co(ii)-4-CNpy metalloporphyrins (1–2) in the catalytic oxidation of the methylene blue dye (MB) were investigated at room temperature in aqueous solution both in the absence and in the presence of hydrogen peroxide. The adsorption spectrum of MB in absence of hydrogen peroxide as a function of time in presence of complexes 1–2 are given in Fig. 13.

**Fig. 14:**
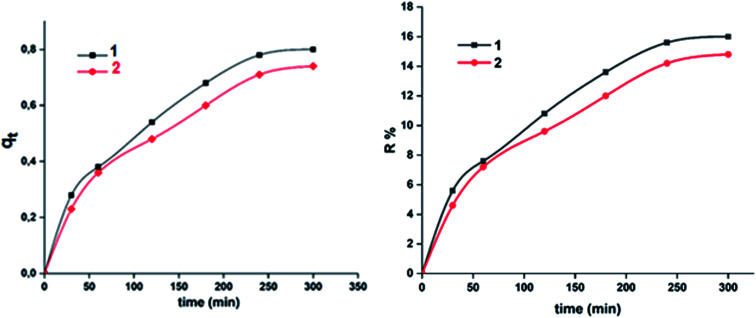
Variation of the adsorption capacity (*q*_*t*_) (left) and the yield removal yield (*R*%) (right) *vs.* time for the MB dye using 1–2.

Fig. 14 illustrates the adsorption capacities (*q*_*t*_) and the decolorization yields (*R*%) of the MB dye in the presence of 1–2 as a function of time under the following experimental conditions: room temperature, *C*_o_ = 10 mg l^−1^ (of the MB dye) at pH = 6. Using the relationship 
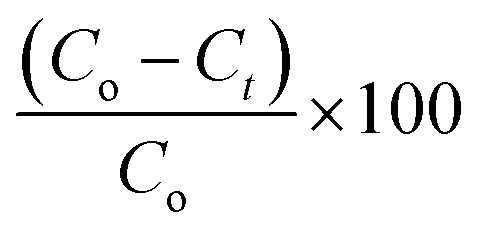
 (*A*_o_ and *A*_*t*_ are the absorption at *t* = 0 and at the *t* instant), the color removal yield values of 1–2 are 16.1% and 14.8%, respectively.

One explanation of the adsorption phenomenon of the MB dye on complexes 1–2, could be the intermolecular interactions occurring between the nitrogen atoms, the methoxy groups and the chlorine atoms present on the surface of the porphyrin complexes 1–2 and the MB organic dye molecule.

The slightly higher *q*_*t*_ and *R*(%) values in the case of the Co-TMPP-4-CNpy derivative (1) compared to those of the Co-TClPP-4-CNpy species (2), could be explained by the fact that for the TMPP derivative (1) the adsorption involves both the C–H bonds and the oxygen atoms of the methoxy groups while for the TClPP species (2) only the chlorine atom of this *meso*-porphyrin is involved (Fig. SI-10†).

**Fig. 15:**
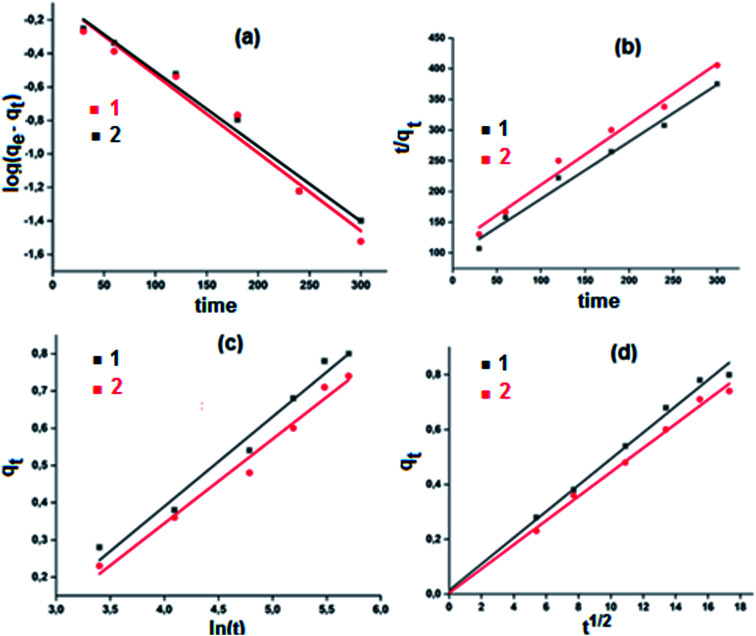
Kinetic data linearized for the adsorption of the MB dye using complexes 1–2. (a) Lagergren pseudo first order model, (b) pseudo second order model, (c) Elovich model and (d) intra-particle diffusion model.

A theoretical kinetic approach was used in order to understand the adsorption process between the MB dye and our two cobaltous derivatives 1–2. This include, the Lagergren pseudo first order, the pseudo second order, the Elovich and the intra-particular diffusion.^71^ The plots corresponding to the four kinetic models are reported in Fig. 15.

The results obtained using the four models which were evaluated based on the regression coefficient *R*^2^ values are given in Table 6. In reference to the high *R*^2^ values, the experimental results are better fitted using the intra-particular diffusion model for both complexes 1–2. Complex 1 exhibits a better fit than that of complex 2.

**Table 6:** Kinetic data for the adsorption of MB dye using complex 1–2 (*C*_o_ = 10 mg l^−1^, pH = 6, *m* = 10 mg)^a^

Kinetic equations	Calculated parameters	1	2
Lagergren pseudo first order	*K*_1_ (min^−1^)	0.142	0.144
	*q* (mg g^−1^)	0.87	0.86
	*R* ^2^	0.98	0.97
Pseudo-second order	*K*_2_ (g mg^−1^ min^−1^)	0.009	0.008
	*q* (mg g^−1^)	1.07	1.01
	*R* ^2^	0.9863	0.9860
Elovich	*α* (mg g^−1^ min^−1^)	0.022	0.019
	β (mg g^−1^ min^−1^)	4.16	4.4
	*R* ^2^	0.9785	0.9825
Intra-particular-diffusion	*K*_d_ (mg g^−1^ min^−1/2^)	0.048	0.044
	*C* (mg g^−1^)	0.994	0.996
	*R* ^2^	0.013	0.004

a *K*_1_: is the pseudo first order kinetic constant (min^−1^), *K*_2_: is the pseudo-second order kinetic constant (g mg^−1^ min^−1^), *K*_d_: is the intra-particular-diffusion kinetic constant (g mg^−1^ min^−1/2^), *α*: is the initial adsorption rate (mg g^−1^ min^−1^), *β*: is the desorption constant (g mg^−1^) during any experiment.

The first step of the adsorption process is usually described by the first order model and then one or more of the three other models are applicable depending mainly on the adsorbent–adsorbate system used. Weber *et al.*,^72^ reported that for well mixed suspensions, rate-controlling step is the intra-particular diffusion model. This model is based on the following equation (eqn (4)):

4 *q*_*t*_ = *K*_d_*t*^1/2^ + *C*

were *K*_d_ is the interparticle diffusion rate constant and *C* is the intercept and which is a constant that represents the thickness of the limited diffusion layer.

The intra-particular diffusion model requires that the corresponding *q*_*t*_*versus t*^1/2^ curve is linear and must pass through the origin.^73^ Therefore, as already mentioned, the intra-particular diffusion model describes our adsorbent–adsorbate system the best.

**Fig. 16:**
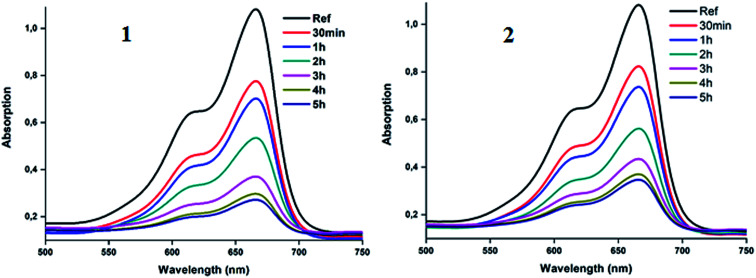
Variation of the *λ*_max_ values of the absorption bands of MB dye and an aqueous H_2_O_2_ solution (4 ml l^−1^ and pH = 6) in the presence of complex 1 (10 mg) (left) and complex 2 (10 mg) (right). The concentration of MB is 10 mg l^−1^ and pH = 6.

In order to improve the ability of the two cobaltous-4-CNpy porphyrins to catalyze the color degradation of the MB dye, the addition of an aqueous hydrogen peroxide solution to the previous used MB dye-1–2 systems was investigated. As expected,^74^ in the presence of the H_2_O_2_ aqueous solution alone (4 ml l^−1^) without the addition of our two Co(ii) coordination compounds, no degradation of the MB dye was observed (Fig. 16). Thus, for a dye concentration of 10 mg l^−1^ and a pH = 6, the color degradation yields are 80% and 78% in the presence of complexes 1–2, respectively. These values are much higher than those obtained without the use of the hydrogen peroxide as an oxidant reagent were the degradation yield values are 16.1% and 14.8% for 1–2, respectively (Fig. 16). Complex 1 gives better catalytic results than complex 2.

**Fig. 17:**
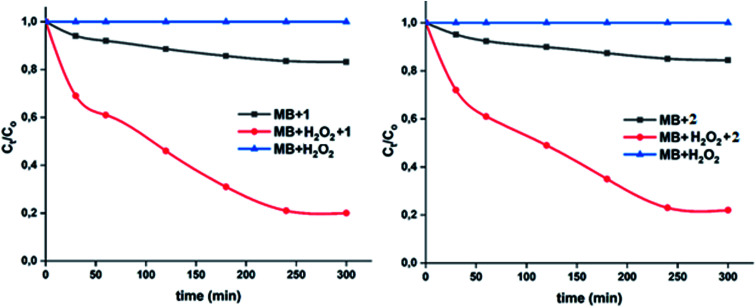
Variation of *C*_*t*_/*C*_o_ as a function of time for the three cases: (i) MB dye and the cobaltous complex, (ii) MB dye with H_2_O_2_ and our Co(ii) complex and (iii) the MB dye with H_2_O_2_ without 1–2. Left: case when complex 1 was used, right: case when complex 2 is used.

The important role of the use of the H_2_O_2_ could be explained by the activation of this oxidant species by the 4-CNpy cobaltous porphyrins complexes 1–2. The plots corresponding to the change of the *C*_*t*_/*C*_o_*versus* time in the case of the MB dye with H_2_O_2_ only, the dye with complexes 1–2 and the dye with 1–2 and H_2_O_2_ are given in Fig. 17.

**Fig. 18:**
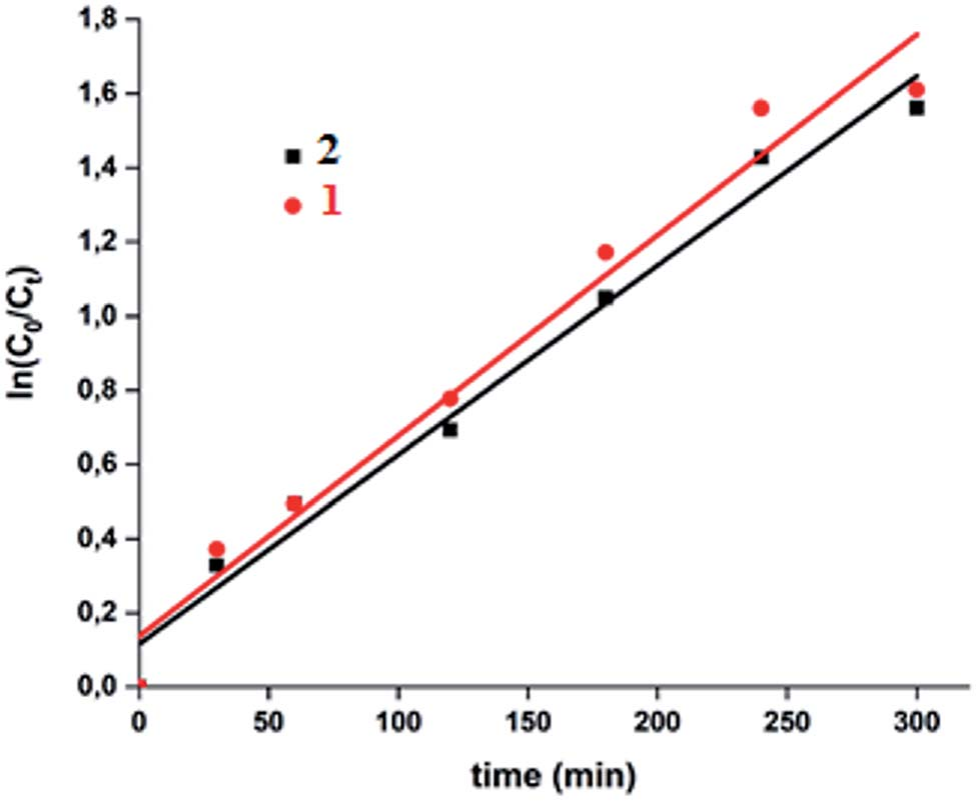
Fitting data of the intra-particular-diffusion curves for the “MB dye-H_2_O_2_-complexes 1–2” systems.

We calculated the kinetic constants of degradation of the MB dye (*K*_o_) when 1–2 are used in the presence of the H_2_O_2_ using the following equation (eqn (5)):^75^5
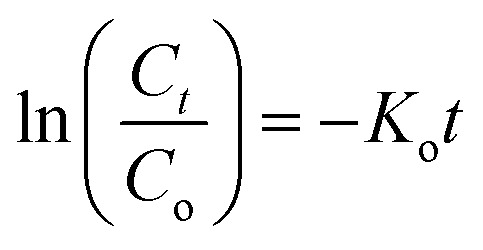
where *C*_*t*_ and *C*_o_ being the MB dye concentration at times *t* and 0, *t* is the time of degradation and *K*_o_ is the first order rate constant. The values of *K*_o_ deduced from Fig. 18 are 0.0054 (with *R*^2^ = 0.97) and 0.0051 (with *R*^2^ = 0.98) for 1–2, respectively.

## 4. Conclusion

We have successfully synthesized two new 4-cyanopyridine cobaltous coordination compounds using two *meso*-phenylporphyrins substituted in the *para*-phenyl positions by the strong donor character methoxy group (H_2_TMPP porphyrin) and the chlorine atom (H_2_TClPP porphyrin) known to have a medium withdrawing character. The [Co^II^(TMPP)(4-CNpy)] (complex 1) and [Co^II^(TClPP)(4-CNpy)] (complex 2) were characterized by UV-visible, IR, ^1^H NMR, ESI-MS and cyclic voltammetry techniques. The UV-visible and the proton NMR as well as the ESI-HRMS, for complex 1 and ESI-MS for complex 2 confirm the coordination in solution of the 4-cyanopyridine leading to a pentacoordinated cobaltous metalloporphyrin, while the ^1^H NMR data show that 1–2 are paramagnetic low-spin (*S* = 1/2) Co(ii) porphyrin complexes. All these investigations indicate that neither the nature of the *para*-substituted group at the phenyls of the *meso*-porphyrin (donor or withdrawing characters) nor the strong π-acceptor character of the 4-cyanopyridine axial ligand have an important role on the electronic properties of 1–2 in solution. This is very similar to the other already known Co(ii) metalloporphyrins, but different in solid state as shown by the X-ray molecular structures of 1–2. In fact, the average equatorial distance between the center metal and the nitrogens of the pyrrole rings (Co–N_p_) of the TMPP derivative (1) is much shorter than that of the TClPP cobaltous species (2). This could be explained by the important ruffling of the porphyrin core of 1 due to the important donor character of the OMe group in the *para*-phenyl position of the TMPP porphyrinate which destabilizes the π orbitals of the porphyrin core and therefore leads a high ruffling deformation of the porphyrin macrocycle. On the other hand, the TClPP-Co(ii) derivative (2) exhibits a short metal–metal distance with a Co⋯Co distance of 5.663 Å compared to the 8.636 Å distance found in the case of the TMPP-Co(ii) species (1). Hence, the [Co^II^(TClPP)(4-CNPy)] (2) in the solid state forms supramolecular dimers. The existence of these dimers could be explained (i) by the absence of a bulky group such as the OMe group in the *para*-positions of the phenyls as is the case for [Co^II^(TMPP)(4-CNpy)] (1) and (ii) by the electron withdrawing effect of the Cl instead of the OMe on the *para*-positions of the phenyls of TClPP and TMPP porphyrinato, respectively, which causes a more efficient π–π interaction leading to a shorter Co⋯Co distance for the Co(ii)-TClPP complex. The formation of such supramolecular dimers in the structure of 2 is a magnetically important consideration for future SQUID and RPE studies on both 1–2. The ability of 1–2 to interact with the methylene blue dye (MB) at room temperature in aqueous solution and pH = 6 was investigated. The kinetic modeling was best fitted along the intra-particular-diffusion and the dye removal yields, without H_2_O_2_ are 16.1% and 14.8% for complexes 1–2, respectively. When a hydrogen peroxide aqueous solution was added to the “MB dye–complexes 1–2” system, the degradation of the dye reached 80% and 78% for 1 and 2, respectively.

## Conflicts of interest

There are no conflicts to declare.

## Supplementary Material

RA-010-C9RA08504A-s001

RA-010-C9RA08504A-s002
