# Supplementary material for: Effect of the coordination of π-acceptor 4-cyanopyridine ligand on the structural and electronic properties of meso-tetra(para-methoxy) and meso-tetra(para-chlorophenyl) porphyrin cobalt(ii) coordination compounds. Application in the catalytic degradation of methylene blue dye
Source: RSC Adv. 2020 Feb 14;10(12):6900–18. doi: 10.1039/c9ra08504a (PMC9049721; doi:10.1039/c9ra08504a)
Supplement: RA-010-C9RA08504A-s001 [file RA-010-C9RA08504A-s001.pdf]

Electronic Supplementary Material (ESI) for RSC Advances.
This journal is © The Royal Society of Chemistry 2020

Effect of the coordination of the π-acceptor 4-cyanopyridine ligand on the structural and electronic properties of the *meso*-tetra(*para*-methoxy) and the *meso*-tetra(*para*-chlorophenyl) porphyrins cobalt(II) coordination compounds. Application in the catalytic degradation of the methylene blue dye

Table of contents

1. Mass spectrometry …………………………………………………..page 2

2. ^{1}H NMR spectra ……………………………..………………….page 3

3. Fluorescence spectroscopy ………………………………………page 4

4. Crystallographic data ……………………………………………page 5

5. The catalytic Adsorption and degradation of the MB dye ……..page 11

1. Mass spectroscopy

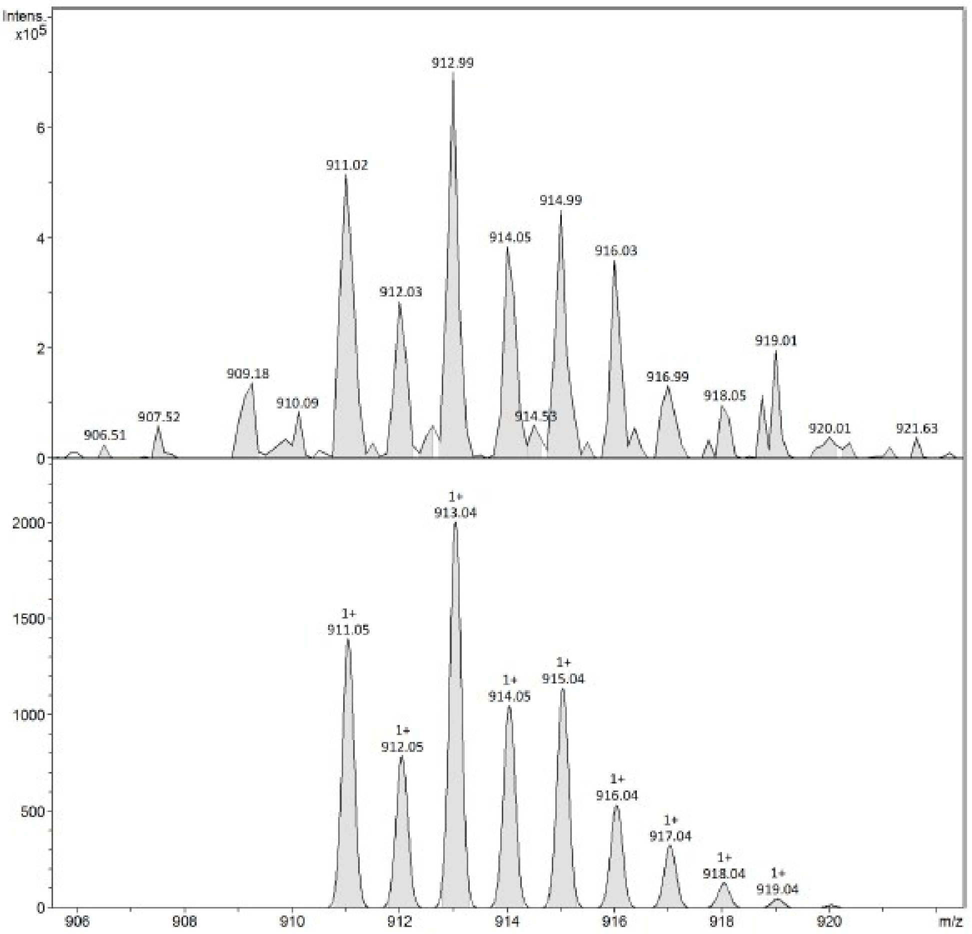

Figure SI-1. ESI- spectrum of complex **2** showing the isotopic ratio in m/z = 913 region ([Co^{II}(TClPP)(4-CNpy)]$^{+}$). The solvent used is the THF with a concentration of 5.10^{-3}M and diluted in methanol with a concentration of 5.10^{-5} M.

2. ^{1}H NMR spectroscopy

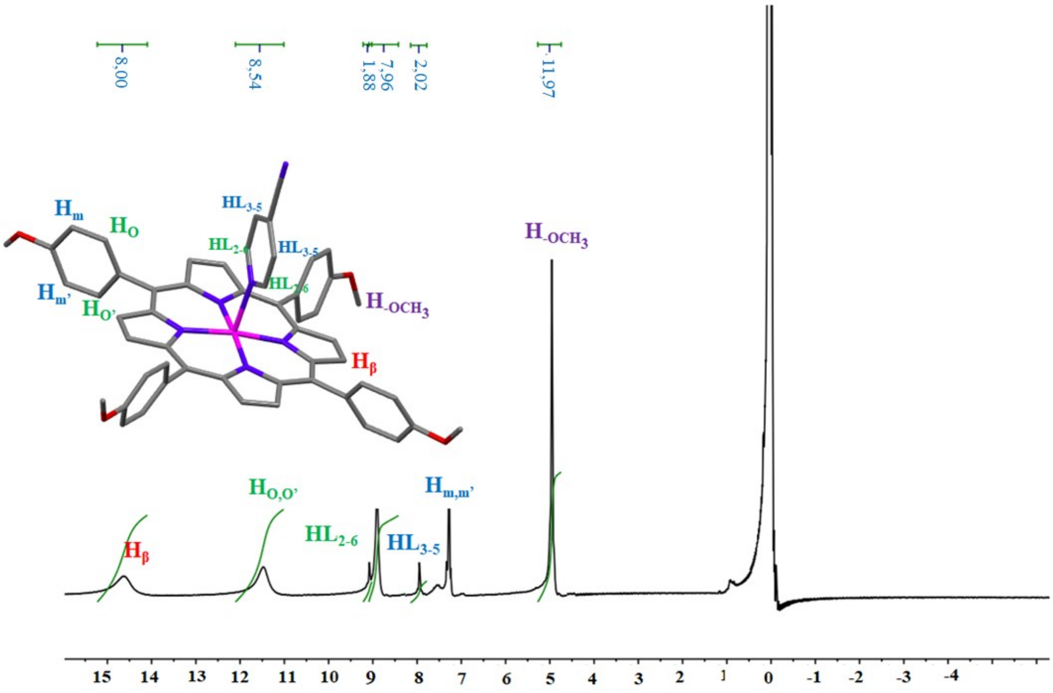

Figure SI-2. ^{1}H NMR (400 MHz) spectra in $CDCl_3$ of complex [Co^{II}(TMPP)(4-CNpy] (**1**). The concentration is ~ 10^{-3} M.

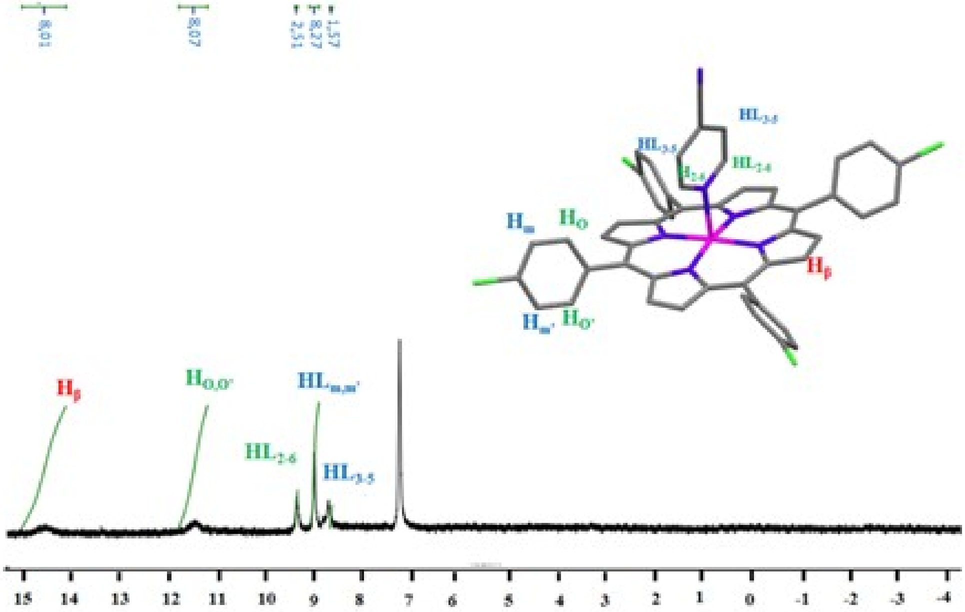

Figure SI-3. ^{1}H NMR (400 MHz) spectra in $CDCl_3$ of complex [Co^{II}(TClPP)(4-CNpy] (**2**). The concentration is ~ 10^{-3} M.

3. Fluorescence spectroscopy

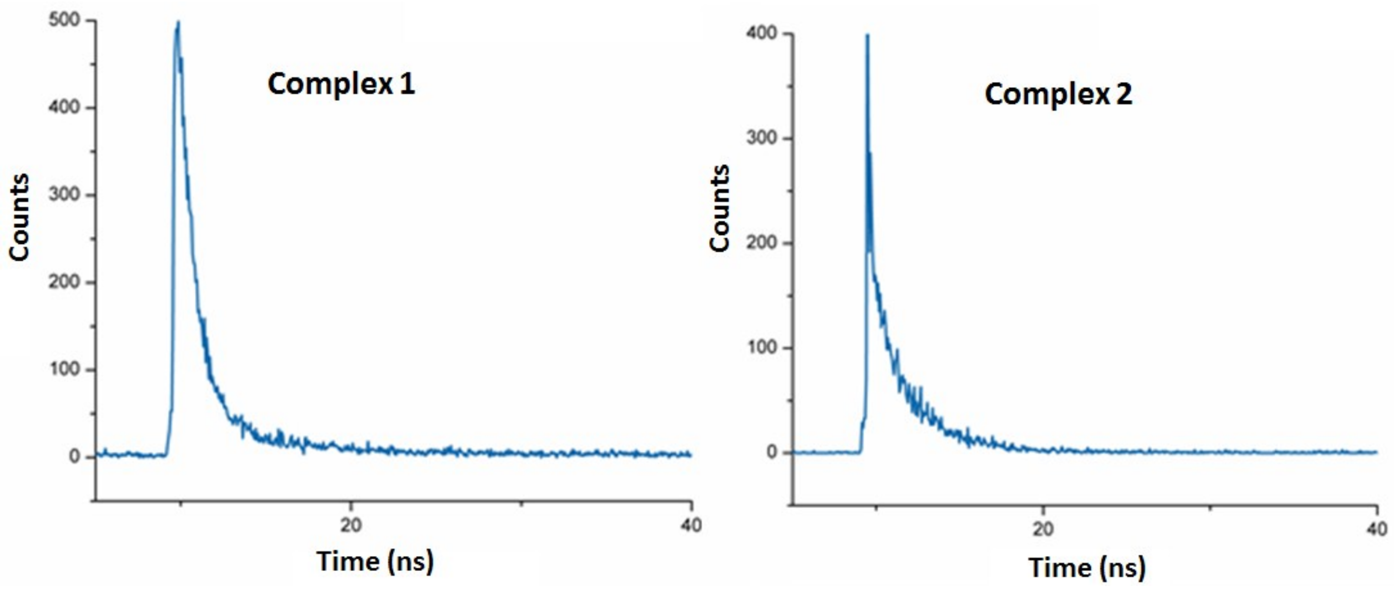

Figure SI-4. Fluorescence decay profiles of [Co^{II}(TMPP)(4-CNpy)] (**1**) (left) and [Co^{II}(TClPP)(4-CNpy)] (**2**) (right).

4. X-ray crystallography

Table SI-1. Selected bond distances (Å) and angles (°) in the molecular structures of complexes **1-2**.

	Complex **1**		Complex **2**
	Cobaltous coordination polyhedron		
Co-N1	1.987(3)	Co-N1	1.968(2)
Co-N2	1.977(3)	Co-N2	1.976(2)
Co-N3	1.984(3)	Co-N3	1.983(2)
Co-N4	1.990(3)	Co-N4	1.980(2)
Co-N5	2.209(3)	Co-N5	2.196(3)
N1-Co-N2	90.21(12)	N1-Co-N2	89.57(10)
N1-Co-N3	171.05(12)	N1-Co-N3	175.71(10)
N1-Co-N4	89.51(12)	N1-Co-N4	89.85(10)
N2-Co-N3	89.21(12)	N2-Co-N3	90.15(10)
N3-Co-N4	90.28(12)	N3-Co-N4	89.67(11)
N2-Co-N4	174.92(11)	N2-Co-N4	169.84(11)
N5-Co-N1	94.77(11)	N5-Co-N1	94.24(9)
N5-Co-N2	91.96(11)	N5-Co-N2	94.55(10)
N5-Co-N3	94.17(11	N5-Co-N3	90.05(10)
N5-Co-N4	93.11(11)	N5-Co-N4	95.62(10)

4-cyanopyridine axial ligand			
N5-C49	1.340(5)	N5-C45	1.338(4)
C49-C50	1.370(5)	C45-C46	1.384(5)
C50-C51	1.387(5)	C46-C47	1.379(5)
C51-C52	1.378(5)	C47-C48	1.392(5)
C52-C53	1.390(5)	C48-C49	1.382(5
C53-N5	1.332(4)	C49-N5	1.326(4)
C51-C54	1.449(5)	C47-C50	1.446(5)
C54-N6	1.139(5)	C50-N6	1.146(5)
C49-N5-Co	120.9(2)	C45-N5-Co	121.4(2)
C53-N5-Co	122.0(2)	C49-N5-Co	120.7(2)
C49-N5-C53	117.0(3)	C45-N5-C49	117.7(3)
C50-C51-C54	119.5(4)	C46-C47-C50	121.5(3)
C52-C51-C54	121.3(3)	C48-C47-C50	119.6(3)
C51-C54-N6	178.7(5)	C47-C50-N6	179.1(5)

Table SI-2**.** Crystal data and structural refinement for (**2**), [Co^{II}(TMPP)(4,4'-bipy)].$CHCl_3$ (**1**) and [Co^{II}(TClPP)(4,4'-bipy)] (**2**).

	Complex **1** TMPP-4-CNpy	Complex **2** TClPP-4-CNpy
Formula	$C_{55}H_{41}N_6O_4Cl_7Co$	$C_{50}H_{28}N_6Cl_4Co$
Crystal System	Monoclinic	Triclinic
Crystal	*C2/c*	*P-1*
a (Å)	36.676 (4)	11.081(2)
b (Å)	13.413 (2)	13.386(3)
c (Å)	19.944 (2)	15.426(3)
α (°)	90.00	83.38(3)
β (°)	103.378 (4)	83.88(3)
γ (°)	90.00	88.67(3)
V (Å^3)	9544.6(18)	2259.8(8)
Z	8	2
$\rho_{calc.}$/ g cm^{-3}	1.413	1.343
X-ray radiation	Mo Kα	Mo Kα
μ/ mm^{-1}	0.583	0.658
F(000)	4184	930
Crystal size (mm^3)	0.56 x 0.31 x 0.07	0.40 x 0.30 x 0.29
Crystal Color	dark	black
Crystal Shape	prism	block
T (K)	150 (2)	200 (2)
θ_{min} - θ_{max} (°)	3.038 – 25.998	1.915 – 25.999
Limiting indices	$-40 \leq h \leq 40$, $-16 \leq k \leq 15$, $-14 \leq l \leq 24$	$-13 \leq h \leq 13$, $-16 \leq k \leq 16$, $-18 \leq l \leq 19$
R(int)	0.0413	0.0527
Reflections collected/unique	39202 / 9176	30240 / 8766
Observed data [$I_o > 2\sigma(F_o)$]	7380	6899
Parameters/Rest	640 / 75	587 / 0
S [all data]	1.033	1.131
R_1^a, wR_2^c [$F_o > 4\sigma(F_o)$]	R_1 <= 0.0683, wR_2 = 0.1695	R_1 = 0.0638, wR_2 = 0.1801
[all data]	R_1 = 0.0860, wR_2 = 0.1847	R_1 = 0.0821, wR_2 = 0.2025

Min./max. res. (eÅ^{-3})	0.977 / -0.878	0.822 / -0.951
CCDC	1950659	1950658

^{a}: $R1 = \Sigma||F_o| - |F_c||/\Sigma|F_o|$, ^{b}: $wR2 = \{\Sigma[w(|F_o|^2 - |F_c|^2)^2]/\Sigma[w(|F_o|^2)^2]$

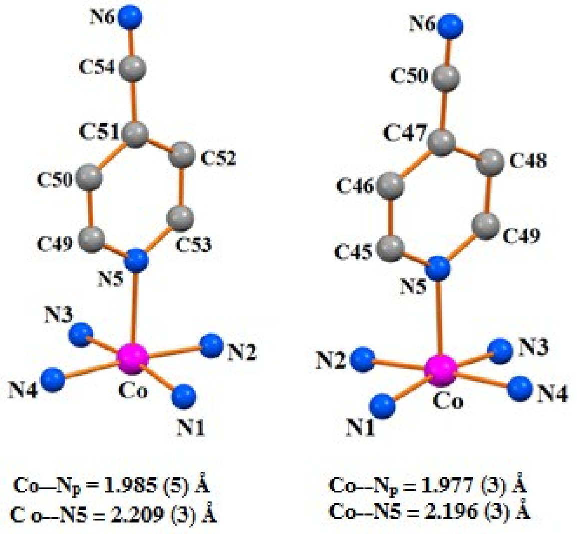

Figure SI-5. Coordination polyhedra of the cobalt center metal in [Co^{II}(TMPP)(4-CNpy)] (**1**) (left) and In [Co^{II}(TClPP)(4-CNpy)] (**2**) (right).

Table-SI-3. Selected hydrogen bonds for complexes **1-2**.

D –H···A[a]	Symmetry of A	D···A (Å)	D–H···A (°)
	Complex **1**		
C8-H8-O2A	1-x,1-y,2-z	3.325(4)	147
C27A-H27C-Cl3B	x-1/2,3/2-y,z+1/2	3.219(13)	154
C49-H49-N4	x,y,z	3.121(5)	121
C53-H53-N2	xyz	3.103(5)	120
C55B-H55-N2	x,y,-1+z	3.199(7)	143
C52-H52-N6	3/2-x,3/2-y,2-z	3.544(9)	136
C3-H3-CL3B	3/2-x,1/2+y,1/2-z	3.521(9)	124
C8-H8-O2B	1-x,1-y,2-z	3.5325(5)	147
C12-H12-O1A	1-x,y-1,3/2-z	3.769(11)	141
C13-H13-CL3A	3/2-x,1/2-y,1-z	3.412(6)	134
C48-H48A-O3A	3/2-x,1/2-y,1-z	3.431(7)	142
C48-H48A-O3B	3/2-x,1/2-y,1-z	3.431(7)	142
C17-H17-N6	3/2-x,y-1/2,3/2-z	3.579(6)	129
C22-H22-Cl1A	3/2-x,3/2-y,1-z	3.806(6)	144
C22-H22-Cl1B	3/2-x,3/2-y,1-z	3.806(6)	144
C26-H26-O2A	x,1-y,z-1/2	3.707(5)	165
C26-H26-O28	x,1-y,z-1/2	3.707(5)	165
	Complex **2**		
C48-H48-N6	1-x,1-y,2-z	3.527(6)	156

[a] : D = donor atom and A = acceptor atom.

Table-SI-4. Selected intermolecular C–H...Cg interactions of complexes **1-2**.

D –H···A[a]	Symmetry of A	D···A (Å)	D–H···A (°)
	Complex **1**		
C27A-H27A-Cg12	1-x,1+y,3/2-z	3.719(9)	139
C30A-H30A-Cg1	x,1-y,1/2+z	3.680(11)	131
C30B-H30B-Cg1	x,1-y,1/2+z	3.619(10)	125
C34A-H34C-Cg4	1-x,y,3/2-z	3.463(15)	115
C46-H46-Cg3	x,1-y,z-1/2	3.647(4)	134
C48-H48B-Cg14	3/2-x,3/2-y,1-z	3.664(6)	153
C56-H53-Cg11	1-x,1+y,3/2-z	3.563(4)	147
C27B-H27D-Cg11	1-x,1+y,3/2-z	3.659(12)	126
C27B-27F-Cg3	x,1+y,z	3.536(14)	120
C41B-H41D-Cg9	x,y-1,z	3.36(3)	113
C41B-H41F-Cg1	x,y-1,z	3.7(3)	159

Complex **2**

C48-H48-N6	-x+1,-y+1,-z+2	3.527 (6)	155
C23-H23-Cg1	1-x,1-y,1-z	3.613(4)	137

[a] : D = donor atom and A = acceptor atom.

Complex 1

- *Cg*1 is the centroid of the N1/C(1)-C(4) phenyl ring.
- *Cg*3 is the centroid of the N3/C(11)-C(14) phenyl ring.
– *Cg*4 is the centroid of the N4/C(16)-C(19) phenyl ring.
– *Cg*9 is the centroid of the N5/C(49)-C(53) phenyl ring.
– *Cg*11 is the centroid of the C(28A)-C(33A) phenyl ring.
– *Cg*12is the centroid of the C(28B)-C(33B) phenyl ring.
– *Cg*13 is the centroid of the C(35)-C(40) phenyl ring.
– *Cg*14 is the centroid of the C(42)-C(47) phenyl ring.

Complex 2

- *Cg*1 is the centroid of the N1/C(1)-C(4) phenyl ring.

Table-SI-5. Intermolecular interactions type Cg…Cg[a,] for complex **2**.

Cg(I)[a]	Cg(J)[a]	Cg…Cg[a]	α[a]	CgI_Perp[a]	CgJ_perp[a]
Cg(1)[b]	Cg2[c]	3.785(2)	15.1(2)	3.397(1)	3.614(1)

[a]: *Cg…Cg* = disytance brtween ring centroids, *α* = dihedral angle between planes *I* and *J*, *CgI_Perp* = perpendicular distance of *Cg(I)* on ring *J*, *CgJ_Perp* = perpendicular distance of *Cg(J)* on ring *I*.
[b]: *Cg1* = the centroid of the N1/C(1)-C(4) phenyl ring.
[c]: *Cg2* = the centroid of the N2/C(6)-C(9) phenyl ring.
Symmetry code: -x,1-y,1-z

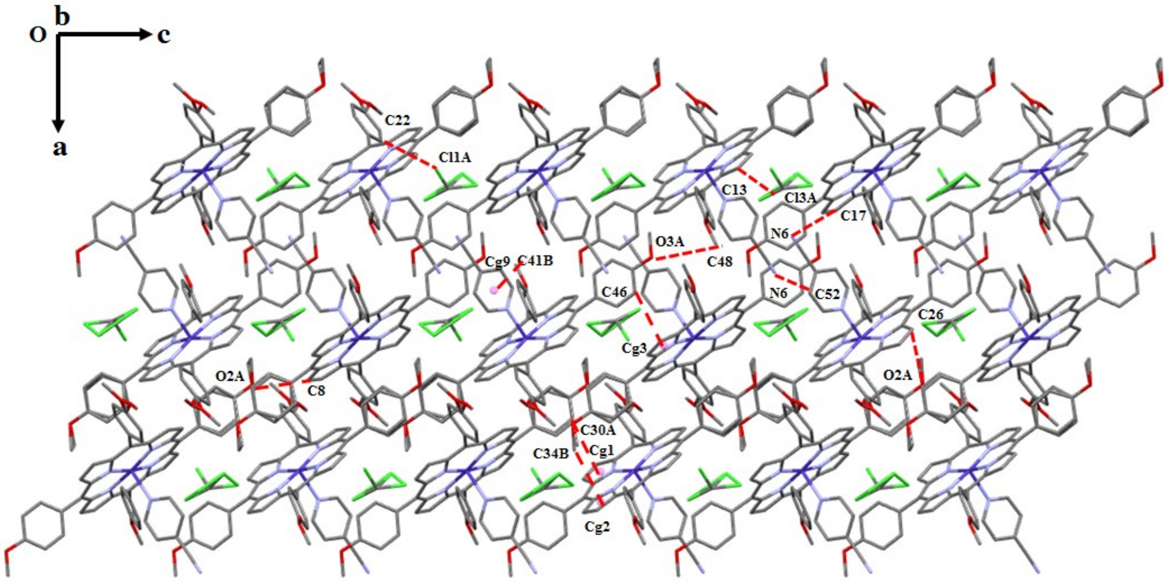

Figure SI-6. The crystal structure of 1 showing the chloroform solvent molecules occupying the voids parallel to the [010] direction.

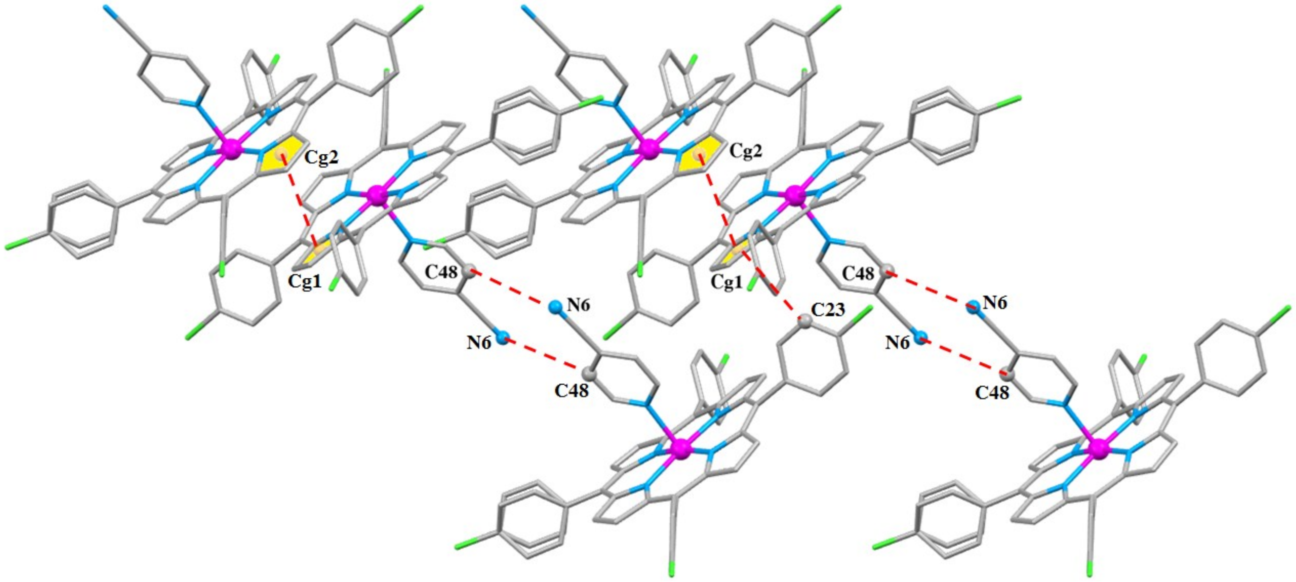

Figure SI-7. Schematic representation showing the weak C—H···N6 intramolecular hydrogen, weak C—H···Cg end Cg…Cg π-π intermolecular interactions in complex 2.

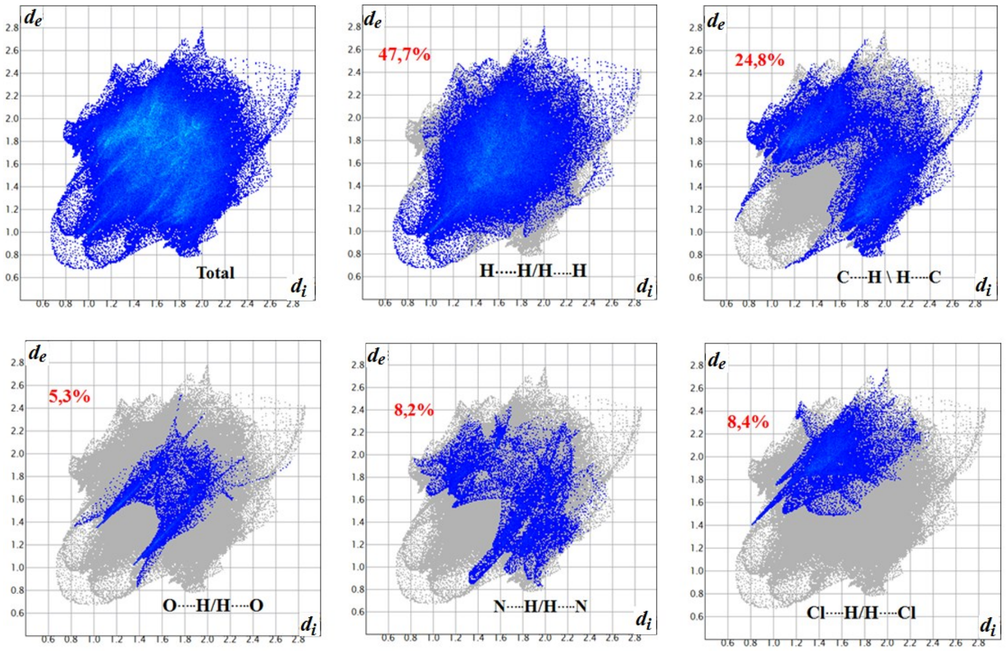

Figure SI-8. 2D fingerprint plots of complex **1** with the major decomposition plots.

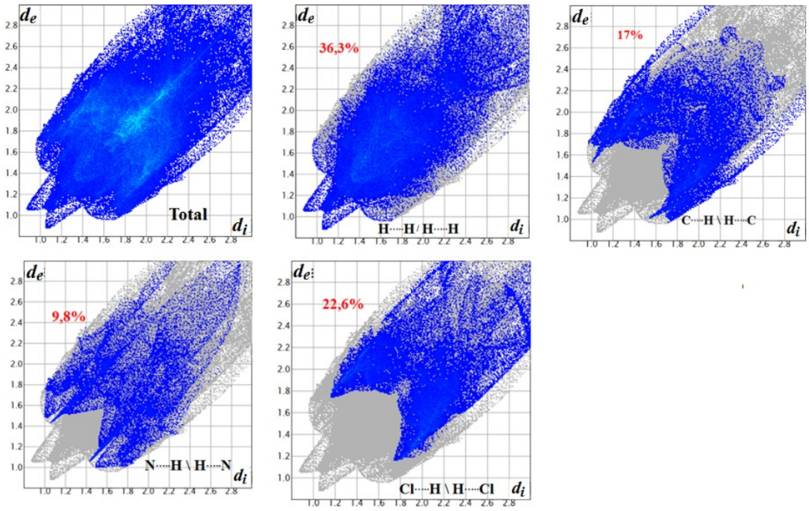

Figure SI-9. 2D fingerprint plots of complex **2** with the major decomposition plots.

5. The catalytic Adsorption and degradation of the MB dye

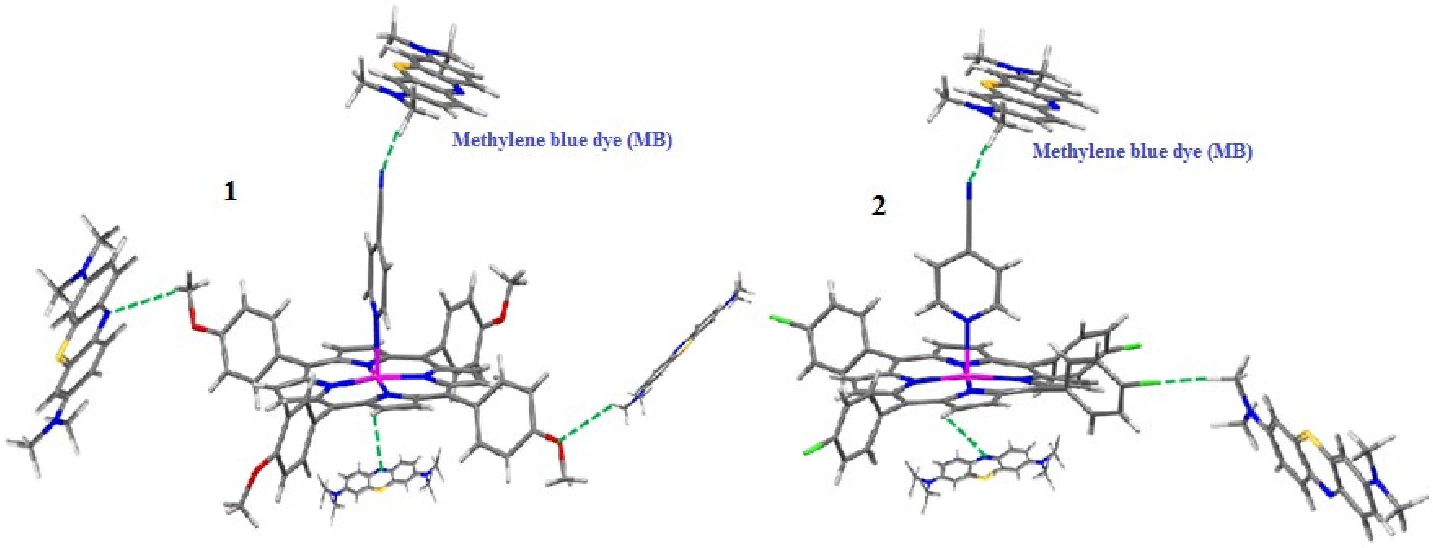

Figure SI-10. Schematic representation showing the adsorption of the methylene blue dye molecules on complexes **1-2**.
